# Impact of smoking on subtypes and molecular profile of breast cancer: a systematic review

**DOI:** 10.37349/etat.2026.1002376

**Published:** 2026-06-16

**Authors:** Georgia Ntella, Anastasia Bothou, Giannoula Kyrkou, Anna Deltsidou, Vasiliki E. Georgakopoulou, Maria Tzeli, Maria Vlachou, Athina Diamanti

**Affiliations:** IRCCS Istituto Romagnolo per lo Studio dei Tumori (IRST) “Dino Amadori”, Italy; ^1^Department of Midwifery, Faculty of Health and Care Sciences, University of West Attica, 12243 Athens, Greece; ^2^Department of Pathophysiology, Laiko General Hospital, National and Kapodistrian University of Athens, 11527 Athens, Greece

**Keywords:** breast cancer, cigarette smoking, molecular subtypes, epigenetics, prognosis

## Abstract

**Background:**

Breast cancer encompasses heterogeneous pathological and molecular subtypes with distinct aetiologies and clinical outcomes. Although cigarette smoking is an established carcinogenic exposure, its subtype-specific associations and molecular effects in breast cancer remain insufficiently clarified. This systematic review synthesizes epidemiological, molecular, and prognostic evidence on how active cigarette smoking influences the risk of specific breast cancer subtypes.

**Methods:**

We conducted a systematic review, including observational and translational human studies that assessed active cigarette smoking in relation to breast cancer subtypes defined by estrogen receptor (ER), progesterone receptor (PR), and human epidermal growth factor receptor 2 (HER2) status, or by intrinsic molecular classifications. We also included studies evaluating smoking-associated molecular alterations within breast tumors.

**Results:**

Nineteen studies met the eligibility criteria. Epidemiological evidence suggested a possible modest increase in the risk of luminal/ER-positive breast cancer, particularly among women with longer smoking duration, heavier cumulative exposure, or smoking initiation before first full-term pregnancy; however, the pooled meta-analysis for current vs. never smoking was not statistically significant. No meaningful association was identified for triple-negative breast cancer (TNBC), and findings for HER2-positive breast cancer were heterogeneous. Molecular studies were associated with smoking-related changes in promoter DNA methylation, higher overall mutational burden, increased genomic instability, altered immune-cell infiltration within the tumor microenvironment, and conversion of receptor phenotype—especially toward HER2 positivity—suggesting a potential association with more aggressive tumor characteristics. Prognostic studies generally showed poorer overall survival and a higher risk of disease recurrence among smokers.

**Discussion:**

Active cigarette smoking may be associated with a possible modest increase in the risk of luminal/ER-positive breast cancer, while being associated with molecular alterations linked to more aggressive tumor phenotypes and poorer clinical outcomes.

## Introduction

Breast cancer is the most frequently diagnosed malignancy in women and a leading cause of cancer death, accounting for about one in four female cancer cases despite advances in screening and therapy [1, 2]. Global incidence is around 48 per 100,000 women, with the highest rates in Western Europe, North America, Australia and New Zealand, and a rising burden in many low- and middle-income countries [1, 2].

Biologically, breast cancer comprises distinct entities defined by gene-expression patterns and key biomarkers. Intrinsic molecular subtypes [luminal A, luminal B, human epidermal growth factor receptor 2 (HER2)-enriched, basal-like] show different prognoses, treatment responses, and risk factor profiles [3, 4]. Large multi-omics projects such as The Cancer Genome Atlas (TCGA) confirmed at least four major genomic classes and substantial inter- and intra-tumors heterogeneity [5]. In routine practice, immunohistochemistry for estrogen receptor (ER), progesterone receptor (PR), HER2, and Ki-67 is used to classify tumors into luminal A-like, luminal B-like, HER2-positive and triple-negative breast cancer (TNBC), categories with important prognostic and therapeutic implications [5].

Established risk factors include reproductive history, hormonal exposures, family history and genetic susceptibility, obesity, and alcohol intake [6]. The role of active cigarette smoking has been more controversial, but updated evaluations from the International Agency for Research on Cancer (IARC) now consider tobacco smoking causally related to breast cancer based on accumulating epidemiological evidence [7]. Meta-analyses indicate that ever-smokers have a modestly increased risk compared with never-smokers, particularly with early initiation (before first full-term pregnancy) and high cumulative exposure, with clear dose-response patterns for duration and intensity of smoking [8–11].

Evidence increasingly suggests that smoking-breast cancer associations vary by tumor subtype. Intrinsic subtypes differ in prognosis and in their risk factor profiles, supporting distinct aetiologic pathways [3, 4, 6]. Large cohort and nested case-control studies examining ER/PR/HER2-defined subtypes generally report stronger associations of smoking with hormone-receptor-positive or luminal-like tumors, with weaker or inconsistent associations for HER2-enriched tumors and TNBC [9, 10, 12–14]. Other population-based data show more complex patterns, including divergent effects by menopausal status, smoking duration and intensity, and modification by body mass index, particularly for HER2-positive disease [9, 10, 12–14].

Beyond incidence, there is growing interest in how smoking may influence tumor biology and outcomes. Experimental and translational work suggests that tobacco smoke carcinogens can reach the breast, induce DNA damage, promote epithelial-mesenchymal transition and stem-like phenotypes, and modulate immune and stromal microenvironments, potentially favoring more aggressive behavior [14–17]. Clinico-pathological series report variable associations between smoking and tumor characteristics or prognosis, with some studies showing no major impact in early hormone-receptor-positive disease [18], while others describe higher frequencies of triple-negative tumors, more severe molecular/stage profiles, and worse short-term survival in smokers, as well as higher rates of HER2 conversion at recurrence [15, 16]. Multi-omics analyses have identified smoking-related differences in mutational burden, copy-number alterations, immune infiltration, and gene-expression signatures, and proposed smoking-associated prognostic models [17].

Most previous reviews and meta-analyses have focused on overall breast cancer incidence in relation to tobacco exposure, often pooling all tumor types and emphasizing active vs. passive smoking [8–10]. The degree to which active smoking preferentially affects specific pathological or molecular subtypes, and its links to distinct molecular profiles, receptor conversion, or multi-omic signatures, has not been systematically synthesized. In addition, the modifying roles of menopausal status, adiposity, and other host factors, and the integration of epidemiologic findings with translational data on tumor biology, remain incompletely addressed [9, 12, 15, 17].

In this context, a systematic review specifically examining the impact of cigarette smoking on breast cancer subtypes and on the molecular profile of breast tumors is warranted. By combining epidemiological evidence on subtype-specific risks with molecular data on biomarkers, gene-expression patterns, epigenetics, and receptor conversion in relation to smoking, this review aims to clarify whether tobacco use is differentially associated with particular breast cancer entities, to explore plausible biological mechanisms, and to identify gaps for future research and prevention strategies.

## Materials and methods

### Protocol and reporting

The methodology of this systematic review was developed a priori and follows the recommendations of the Preferred Reporting Items for Systematic Reviews and Meta-Analyses (PRISMA) 2020 statement [19]. The protocol prespecified the research question, eligibility criteria, outcomes, and analysis plan and was completed before study selection began. The protocol was registered in the International Prospective Register of Systematic Reviews (PROSPERO; registration number: CRD420251248713).

### Research questions and eligibility criteria

The review addressed the following questions: among humans, what is the impact of cigarette smoking on (a) the risk of developing specific breast cancer pathological or molecular subtypes and (b) the molecular profile of breast cancer tumors and their clinical outcomes?

Eligibility criteria were defined using the Population, Intervention/Exposure, Comparison, Outcomes, Study design (PICOS) framework.

The population of interest was adult women (≥ 18 years) from any geographic region. Studies including both sexes were eligible if breast cancer outcomes were presented separately for women or if data for women could be extracted. Studies solely in men were excluded.

The exposure of interest was active cigarette smoking, assessed as status (never, former, current), intensity (cigarettes per day), duration (years of smoking), cumulative dose (pack-years), age at initiation or cessation, or other quantitative measures. Studies focusing exclusively on passive smoking were eligible only if they also evaluated active smoking, and data for active smoking could be extracted. Studies solely on passive/second-hand smoke were planned to be summarized narratively but not included in the primary quantitative synthesis. Studies of other tobacco products (e.g., cigars, waterpipe, smokeless tobacco, e-cigarettes) were eligible if conventional cigarette smoking was analyzed separately.

The comparison groups were never-smokers or lower categories of smoking exposure, depending on the original study design. For incidence and risk studies, the primary comparison of interest was ever-smokers or categories of smoking exposure vs. never-smokers. For studies restricted to breast cancer cases, the comparison was between smokers and non-smokers in relation to the distribution of tumor subtypes or molecular profiles, and, where available, between exposure categories in terms of prognosis.

The primary outcomes were, first, the risk or odds of breast cancer by pathological or molecular subtype defined using hormone receptor and HER2 status (e.g., ER/PR/HER2), intrinsic subtype (luminal A-like, luminal B-like, HER2-enriched, triple-negative, basal-like), or similar classifications, and second, differences in tumor molecular profile by smoking status among patients with breast cancer. Tumor molecular profile included immunohistochemistry-based markers (ER, PR, HER2, Ki-67, and related panels), gene-expression signatures, DNA methylation or other epigenetic patterns, transcriptomic, genomic, or multi-omics signatures, and receptor conversion or changes in molecular subtype between primary and recurrent disease. Secondary outcomes included breast cancer-specific mortality, overall survival, progression-free or disease-free survival, and patterns of recurrence by smoking status within defined subtypes or molecular profiles.

Eligible study designs were observational human studies, including prospective and retrospective cohort studies, nested case-control and case-control studies, case-case comparisons, and cross-sectional analyses, as well as translational studies that linked smoking exposure with molecular or genomic features in human breast tumor tissue or normal breast tissue. Experimental in vitro or animal studies were not eligible for the main synthesis but were planned to be used qualitatively in the discussion as mechanistic support when they explicitly examined the effect of tobacco smoke or cigarette smoke condensate on breast epithelial or cancer cells. Case reports and case series with fewer than ten participants, narrative reviews, systematic reviews and meta-analyses, editorials, commentaries, conference abstracts without sufficient data, and non-original articles were excluded.

There were no restrictions on the publication date. No explicit language restrictions were applied at the search stage. However, due to feasibility considerations, only studies published in English-language peer-reviewed journals were included in the final synthesis.

### Research strategy

We systematically searched the following electronic databases from inception to November 2025: MEDLINE via PubMed, Embase, Web of Science Core Collection, and Scopus. Grey literature was explored by screening conference proceedings of major oncology and epidemiology meetings [e.g., American Society of Clinical Oncology (ASCO), European Society for Medical Oncology (ESMO), San Antonio Breast Cancer Symposium (SABCS)] for the last five years, when full articles were not already identified in the main databases. Grey literature was explored by screening conference proceedings from major oncology and epidemiology meetings, including the ASCO, the ESMO, and the SABCS, for the last five years. Searches were conducted through conference abstract databases and official meeting websites using keyword combinations related to breast cancer, smoking, and molecular subtypes. Only abstracts reporting original human data with sufficient methodological detail were considered. No additional eligible studies from the grey literature were included in the final synthesis. The reference lists of all included articles and relevant reviews were manually screened to identify additional eligible studies (backward snowballing), and citation tracking of key studies was performed using Web of Science and Google Scholar (forward snowballing).

The search strategy combined controlled vocabulary (e.g., MeSH, Emtree) and free-text terms for breast cancer, smoking, and pathological or molecular subtypes. The PubMed search strategy, which was adapted for other databases with database-specific subject headings and syntax, was developed iteratively with input from a medical information specialist. The PubMed search strategy, which was adapted for other databases with database-specific subject headings and syntax, was developed iteratively with input from a medical information specialist. The full, database-specific search strategies for all sources (PubMed, Embase, Scopus, and Web of Science) are provided in Supplementary material (Part 1).

### PRISMA process

A comprehensive search of electronic databases identified 4,283 records in total, comprising 1,280 from PubMed/MEDLINE, 1,425 from Embase, 890 from Scopus, and 688 from Web of Science. No additional records were identified through grey literature searches or backward and forward citation tracking. After removal of duplicates (*n* = 1672), a total of 2611 unique records underwent title and abstract screening. Screening was performed independently by two reviewers following the eligibility criteria prespecified in the protocol.

During title and abstract screening, 2,412 records were excluded for not meeting the inclusion criteria. The most common reasons for exclusion were absence of smoking exposure assessment (*n* = 1,085), lack of subtype- or molecular-specific breast cancer outcomes (*n* = 973), non-original articles such as reviews or commentaries (*n* = 236), and non-human or purely experimental cell or animal studies (*n* = 118).

A total of 199 full-text articles were retrieved for detailed evaluation, of which 180 were excluded for predefined reasons. The main exclusion categories were failure to report subtype-specific risk estimates or to stratify outcomes by ER/PR/HER2 or intrinsic molecular subtype (*n* = 72), lack of separate assessment of active cigarette smoking as distinct from passive smoking or mixed tobacco exposures (*n* = 34), insufficient or unusable molecular data, such as descriptive pathology reports without quantifiable or comparative molecular analyses (*n* = 29), incompatible study design, including case reports, small case series with fewer than ten participants, conference abstracts without sufficient data, or studies without original human data (*n* = 27), and lack of extractable smoking data, for example where only pooled “tobacco use” without cigarette-specific categories was reported (*n* = 18).

Following full-text screening, 19 studies met all eligibility criteria and were included in the qualitative synthesis.

**Figure 1:**
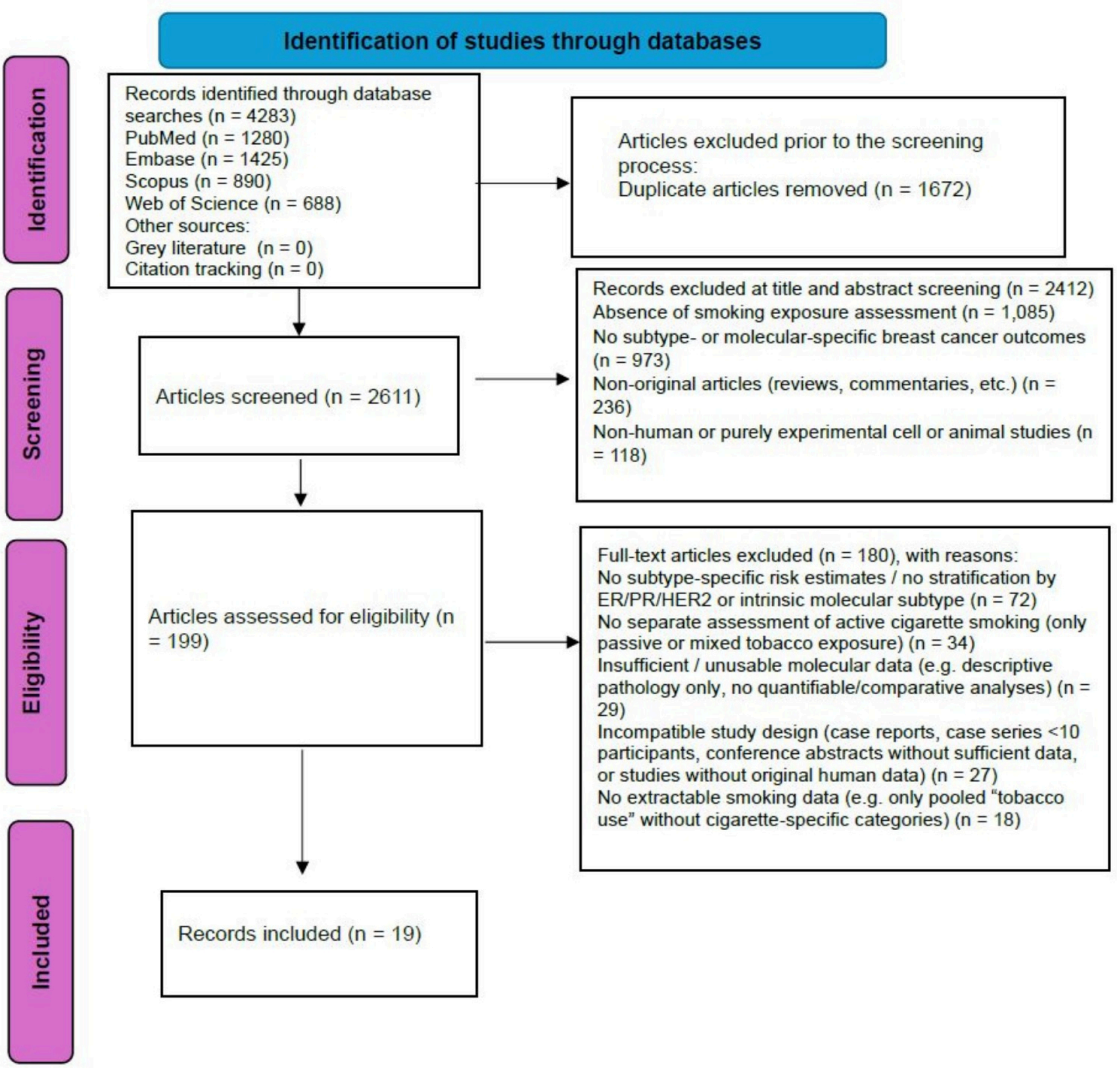
**PRISMA 2020 flow diagram of study identification, screening, eligibility assessment, and inclusion.** Adapted from [19]. © Author(s) (or their employer(s)) 2019. PRISMA: Preferred Reporting Items for Systematic Reviews and Meta-Analyses.

The study selection process is illustrated in Figure 1.

### Data extraction

A standardized data extraction form was developed and piloted on a sample of eligible studies and then refined. Two reviewers independently extracted data from each included study; discrepancies were resolved by consensus, with the involvement of a third reviewer if needed.

For epidemiological incidence or risk studies, we extracted information on: first author, year of publication, country and setting, study design, recruitment period, sample size (cases and controls or cohort size), eligibility criteria, source of controls (for case-control studies), exposure assessment method (self-report, interview, questionnaire, registry), smoking variables (status, intensity, duration, pack-years, age at initiation and cessation, pre- or post-first birth smoking where available), timing of smoking assessment in relation to diagnosis (for cohorts), and adjustment variables included in multivariable models. For tumor characteristics, we extracted the definition and assessment method of pathological and molecular subtypes, including markers used (ER, PR, HER2, Ki-67), cut-offs, classification algorithms (e.g., St. Gallen, PAM50 or similar if available), and the distribution of subtypes by smoking status. For outcomes, we extracted effect estimates comparing smoking categories with never-smokers for overall breast cancer and for each subtype [e.g., odds ratios, relative risks (RRs), hazard ratios] along with their 95% confidence intervals (CIs) and the covariates included in the models.

For studies restricted to breast cancer cases examining tumor molecular profiles, we extracted details on the type of molecular data (immunohistochemistry, gene expression profiling, DNA methylation, genomic or multi-omics), the platforms and assays used, bioinformatics pipelines or classification methods, and how smoking exposure was defined and analyzed (e.g., current vs. never, dose-response categories, continuous pack-years). We collected information on associations between smoking and specific markers (e.g., receptor expression, proliferation indices), molecular signatures, pathway activation, epigenetic patterns, receptor conversion or subtype switching between primary and recurrent disease, and, when reported, survival or recurrence outcomes stratified by smoking status within subtypes or molecular groups.

### Risk of bias assessment

The risk of bias of each included observational study was independently assessed by two reviewers using the Newcastle-Ottawa Scale (NOS), adapted as appropriate for cohort, case-control, or cross-sectional designs [20]. The NOS evaluates three domains: selection of participants, comparability of study groups, and ascertainment of exposure and outcome. Within each domain, studies were awarded stars according to predefined criteria. For cohort studies, higher scores were assigned to studies with clearly defined and representative cohorts, robust smoking assessment, adequate follow-up, and appropriate control for major confounders such as age, reproductive history, alcohol consumption, and body mass index. For case-control studies, emphasis was placed on appropriateness of control selection, matching or adjustment for key confounders, and blinding or standardization of exposure assessment.

For translational and molecular profile studies based on clinical samples or multi-omics datasets, the NOS items were adapted to capture potential sources of bias specific to these designs, such as selection of tumor specimens, completeness and quality of molecular data, and handling of missing smoking information. Disagreements in risk of bias ratings were resolved by discussion.

The results of the risk of bias assessment are available in Table S1.

### Data synthesis and statistical analysis

We anticipated considerable heterogeneity in study design, smoking metrics, subtype definitions, and molecular endpoints. Therefore, our primary approach was a structured narrative synthesis, grouping studies according to their main focus: (1) risk of breast cancer by pathological or molecular subtype in relation to smoking, and (2) tumor molecular profile and clinical outcomes among breast cancer patients according to smoking exposure.

When at least three studies provided sufficiently comparable effect estimates for the same subtype and exposure contrast, we performed quantitative synthesis using random-effects meta-analysis. For incidence and risk studies, we used RRs as reported; when necessary, we transformed effect measures to log RRs and pooled them using inverse-variance weighting. Separate random-effects meta-analyses were conducted for ER-positive breast cancer (current vs. never smoking) and for TNBC (current vs. never and former vs. never smoking). Statistical heterogeneity was evaluated using the Cochran *Q* test and quantified with the *I*^2^ statistic and between-study variance (*τ*^2^). Potential small-study effects and publication bias were to be explored using funnel plots and Egger’s test when at least ten studies contributed to a meta-analysis; in this review, no meta-analysis met this threshold, so these analyses were not performed.

For molecular profile studies, due to the diversity of molecular endpoints and analytic approaches, quantitative pooling was generally not appropriate. Instead, we summarized the direction and strength of associations between smoking and key molecular features (e.g., enrichment of specific gene-expression signatures, methylation changes, receptor conversion) and highlighted consistent patterns across independent datasets.

All statistical analyses were performed using Stata (StataCorp, College Station, TX, USA).

Studies solely on passive/second-hand smoke were planned to be summarized narratively but not included in the primary quantitative synthesis. During screening, a limited number of such studies were identified; however, they were not included in the final review because they did not provide extractable data on active smoking, lacked subtype-specific or molecular outcomes, or were otherwise not aligned with the objectives of the present study

## Results

A total of 19 studies met the inclusion criteria for this review, comprising large prospective cohorts, population-based case-control studies, and hospital-based series from North America, Europe, and South America. Across these studies, breast tumors were classified using routine ER, PR, and HER2 immunohistochemistry into luminal/ER-positive, HER2-positive, and triple-negative subtypes, with some studies further distinguishing luminal A-like and B-like, or using extended IHC panels and multigene signatures [12–18, 21–32]. Smoking exposure was uniformly self-reported and most often captured as ever/former/current status, with several studies also incorporating detailed metrics of intensity, duration, pack-years, and timing of initiation in relation to menarche or first full-term pregnancy. The primary outcomes in the incidence studies were subtype-specific risks of breast cancer, while the molecular and prognostic studies additionally evaluated tumor gene-expression or methylation profiles, receptor conversion, recurrence, and survival endpoints stratified by smoking status.

### Associations between cigarette smoking and breast cancer subtypes

Eight observational studies contributed data on the association between active cigarette smoking and breast cancer risk stratified by pathological or immunohistochemical subtype, most defined by ER, PR, and HER2 status, with some analyses additionally distinguishing basal-like disease [12–14, 21–25] (Table 1). Although the studies differed in design, populations, exposure metrics, and subtype definitions, a broadly consistent pattern emerged: smoking showed a modest positive association with hormone receptor-positive or luminal-type breast cancer, while associations with triple-negative and most HER2-positive tumors were generally weak or absent.

**Table 1:** Overview of study designs and smoking-related variables in included breast cancer subtype analyses.

**Study (author, year)**	**Country/Setting**	**Design, recruitment & follow-up**	**Population & eligibility**	**Sample/Cases**	**Exposure assessment (smoking)**	**Smoking variables used in analyses**	**Timing of exposure assessment**	**Tumor subtype definition & markers**	**Covariates in multivariable models**	**Main smoking-breast cancer associations (vs. never smokers)**
**Kabat et al., 2011 [22]**	USA; multi-centre WHI cohort (clinical trials + OS; 40 centres)	Prospective cohort; recruitment 1993–1998; median follow-up 8.0 years; close-out 12 Sept 2005	Postmenopausal women aged 50–79 years enrolled in WHI CT or OS; excluded if prior breast cancer or mastectomy, or missing key exposure/outcome data	**Cohort analysed:** 148,030 women. **Cases:** TNBC 300; ER+ (HER2 status known) 2,479. **Exclusions:** prior breast cancer/mastectomy 8,735; missing outcome 690; breast cancer without definite ER/PR/HER2 2,263; missing smoking 1,773; missing alcohol 318	Baseline **self-administered questionnaires** (health habits/lifestyle) at WHI entry; smoking was entirely self-reported	Collected: ever smoked ≥ 100 cigarettes; age at initiation; current/former status; age at quitting; cigarettes/day; years smoking. Analysed as: (1) smoking status (never/former/current); (2) cigarettes/day (0–4, 5–14, 15–24, ≥ 25); (3) age at start (< 20, ≥ 20 years); (4) duration (< 20, 20–29, ≥ 30 years); (5) pack-years (< 20, 20–40, ≥ 40), all vs. never	Single baseline assessment at enrolment (1993–1998), **prior to diagnosis**; no repeated updates of smoking were used in analyses	Breast cancer cases self-reported then centrally adjudicated. **TNBC**: ER−/PR−/HER2− (absence of ER and PR expression and no HER2 over-expression). **ER+ breast cancer**: ER positive with known HER2 status. Markers: ER, PR, HER2; Ki-67 not reported. IHC cut-offs not reported; no PAM50 or St Gallen algorithms	Base model: age; age at menarche; age at first full-term pregnancy; parity; age at menopause; BMI; waist circumference; oral contraceptive use; hormone therapy (never, estrogen only, estrogen + progestin, both); history of breast biopsy; family history of breast cancer in first-degree relative; mammogram in past 2 years; physical activity (MET-h/week); education; ethnicity; WHI trial arm or OS component. **Smoking models** additionally adjusted for alcohol intake. **Alcohol models** additionally adjusted for pack-years (0, < 20, 20–40, ≥ 40)	**Smoking status—TNBC (*n* = 300)**: former vs. never HR 0.91 (95% CI 0.70–1.16); current vs. never HR 1.09 (0.69–1.72) → no clear association. **Smoking status—ER+ (*n* = 2,479)**: former vs. never HR 1.14 (1.05–1.24); current vs. never HR 1.05 (0.88–1.25) → modest increase in ER+ risk among former smokers. **Dose metrics (ER+)**: positive trends for cigarettes/day (*p* = 0.02), duration (*p* = 0.03), and pack-years (*p* = 0.01; ≥ 40 pack-years HR 1.24, 95% CI 1.06–1.44), while no significant trends for TNBC (all *p*-trend > 0.3). Baseline distribution by smoking: among TNBC cases, 51.5% never, 41.1% former, 7.4% current; among ER+ cases, 47.8% never, 46.1% former, 6.1% current
**Kawai et al., 2014 [12]**	USA; three-county Seattle-Puget Sound metropolitan area (King, Pierce, Snohomish counties); population-based	Population-based case-control study of women aged 20–44 years; cases diagnosed 2004–2010, identified via SEER Cancer Surveillance System; controls selected by random-digit dialing; interviews generally within ~2 years of reference date	Women 20–44 years of age, resident in the three-county area; cases: incident invasive breast cancer with no prior in situ or invasive breast cancer; controls: cancer-free women frequency matched to cases on 5-year age groups. Exclusions from analysis: ER−/HER2+ (*n* = 60) due to small numbers; missing ER/PR/HER2 (*n* = 28); missing smoking data (5 controls, 8 cases)	Initially, 1,359 eligible cases; 1,056 (78%) interviewed. After exclusions, analytic case set: 960 invasive breast cancer cases (778 ER+, 182 TN). Controls: 1,489 eligible; 943 (63%) interviewed; after excluding 5 with missing smoking data, 938 controls. Final analytic dataset: 938 controls, 778 ER+ cases, 182 ER−/PR−/HER2− (TN) cases	In-person structured interview; detailed lifetime smoking history up to reference date (diagnosis date for cases, assigned reference date for controls). Smoking self-reported. Ever/never based on ≥ 100 cigarettes lifetime; collected ages started/stopped for each period, intensity (cigarettes/day), and recency	Derived variables: (1) ever smoked (never/ever ≥ 100 cigarettes); (2) recency (never/current-recent/former; current-recent = smoking within 2 years of reference date, former = quit ≥ 2 years before reference date); (3) total years smoked (never/< 5.0/5.0–9.9/10.0–14.9/≥ 15.0); (4) age at initiation (never/≤ 14/15–17/≥ 18); (5) pack-years (never/< 2.5/2.5–4.9/5.0–9.9/10.0–14.9/≥ 15.0); (6) years since quitting for former smokers (never/ < 5/5–9.9/≥ 10); (7) initiation before menarche (no/yes); (8) initiation before first birth among parous women (no/yes)	Exposure history was restricted to the period before the reference date; interviews were conducted on average 18 months (cases) and 20 months (controls) after the reference date (medians 16 and 19 months, respectively). Smoking history recalled retrospectively but over a relatively recent time window	ER and PR positivity is defined as ≥ 1% positive staining of tumour cells; negativity is defined as 0–1% positive staining. HER2 positivity is defined as IHC 3+ and/or FISH-positive; HER2 negativity is defined as IHC 0/1+ and/or FISH-negative. Tumours with HER2 IHC 2+ and no FISH result are classified as HER2 unknown. Subtypes: ER+ (all ER+ regardless of PR/HER2); triple-negative (ER−/PR−/HER2−). ER−/HER2+ group excluded due to small numbers	Polytomous logistic regression comparing ER+ and TN cases separately to the common control group. All models adjusted for age (5-year categories) and reference year (continuous). Potential confounders evaluated: education, income, race/ethnicity, oral contraceptive use, mammography history, first-degree family history, BMI, age at menarche, parity, number of full-term pregnancies, age at first live birth, alcohol use, and physical activity. Only age at first live birth changed the risk estimates > 10%, so the final models adjusted for age, reference year, and age at first live birth. No significant effect modification detected	Ever vs. never smoking: overall breast cancer OR 1.3 (95% CI 1.1–1.7); ER+ OR 1.4 (1.1–1.8); TN OR 1.1 (0.7–1.6)—elevation confined to ER+. Current/recent vs. never: ER+ OR 1.4 (1.0–2.0); TN OR 1.2 (0.7–2.1). Former vs. never: ER+ OR 1.4 (1.0–1.8); TN OR 0.9 (0.6–1.5). Pack-years (ever smokers, overall): < 2.5 PY ER+ OR 1.5 (1.1–2.1); ≥ 15 PY ER+ OR 1.7 (1.1–2.5); TN estimates near null, no dose-response. Among current/recent smokers: ≥ 15 years smoking ER+ OR 1.5 (1.1–2.1); ≥ 10 pack-years ER+ OR 1.6 (1.1–2.4); no corresponding increase for TN (OR ≈ 1.0). Years since quitting among former smokers: ER+ risk appeared to return toward baseline ≥ 10 years after cessation. Overall pattern: modest increased risk for ER+, no clear association for TN in young women 20–44.
**Butler et al., 2016 [21]**	USA; 24 adjoining counties in central and eastern North Carolina; Carolina Breast Cancer Study (CBCS) phases I & II	Population-based case-control study. Cases: first primary invasive breast cancer diagnosed 1 May 1993–30 Sept 1995 (Phase I) or 1 May 1996–30 Sept 2001 (Phase II), identified via rapid case ascertainment through the NC Central Cancer Registry. Controls: incidence-density sample, frequency-matched to cases by 5-year age, race, county; controls 20–64 from DMV, ≥ 65 from Medicare. Case response rate 76%, control response 55%.	Female residents of 24-county region, aged 20–74 years, with first diagnosis of invasive breast cancer (cases) or cancer-free at selection (controls). Oversampling of black and younger women to improve power for subtype/race analyses.	Overall analytic set: 1,808 invasive cases and 1,564 controls. Subtype-specific analyses: Luminal cases ≈ 737 (369 never, 368 ever smokers); Basal-like cases ≈ 205 (114 never, 91 ever smokers); controls 1,564 (840 never, 724 ever). Race-stratified counts were reported separately (black: 788 cases/718 controls; white or non-black: 1,020 cases/846 controls).	In-person nurse-administered interview using a standardized questionnaire, typically ~6 months after case ascertainment. Smoking self-reported: lifetime history, age at initiation, age at cessation, number of packs per day. Active smokers were defined as women who had smoked ≥ 100 cigarettes in their lifetime.	Derived variables: (1) ever smoking (never/ever ≥ 100 cigarettes); (2) smoking status (never/former/current)-current = still smoking at interview or quit at same age as case/control selection, former = quit before selection; (3) smoking dose (packs/day: never, < ½, ½–1, > 1); (4) duration (never, ≤ 10, 11–20, > 20 years); duration separately for current and former smokers; (5) years since quitting in former smokers (never, < 5, 5–10, 11–20, > 20); (6) age at initiation (never, ≤ 15, 16–20, > 20 years); (7) initiation relative to menarche and first full-term pregnancy (never; ≤ menarche; after menarche ≥ 11 years before FFTP; after menarche < 11 years before FFTP).	Smoking history up to age at case/control selection; interviews on average ~6 months after diagnosis/selection. Classification of current vs. former explicitly referenced to age at case/control selection to limit reverse causation due to post-diagnosis quitting.	ER and PR positivity was obtained from medical records. Tumour blocks were centrally stained for HER2, HER1, and CK5/6 by IHC in the UNC core lab. Subtypes: luminal = ER+ and/or PR+, regardless of HER2; basal-like = ER−, PR−, HER2−, and HER1+ and/or CK5/6+. Assay procedures and cut-offs for positivity were previously described in CBCS methodological papers.	Unconditional logistic regression with polytomous outcomes (luminal and basal-like vs. common control group). All ORs adjusted for age, race, first-degree family history of breast cancer, alcohol use, menopausal status, hormone replacement therapy use, oral contraceptive use, parity, age at first birth, age at first breastfeeding, age at menarche, BMI, and for randomized recruitment probabilities via offset term. The same adjustment set was applied in overall and subtype-specific models.	Overall breast cancer: Ever vs. never OR 1.07 (95% CI 0.92–1.25); current vs. never OR 1.02 (0.84–1.24); former vs. never OR 1.11 (0.93–1.33). Duration > 20 vs. never OR 1.33 (1.09–1.61); among former smokers, > 20 yrs vs. never OR 1.54 (1.15–2.07); years since quitting 5–10 vs. never OR 1.39 (1.01–1.93). Subtype-specific: ever vs. never—luminal OR 1.12 (0.92–1.36), basal-like OR 0.96 (0.69–1.32). Current vs. never—luminal OR 1.10 (0.86–1.41), basal-like OR 0.82 (0.54–1.24). Duration > 20 yrs vs. never—luminal OR 1.51 (1.19–1.93), basal-like OR 0.90 (0.57–1.43). Dose > 1 pack/day vs. never—luminal OR 1.08 (0.81–1.44), basal-like OR 0.47 (0.25–0.89) (inverse). Tests of heterogeneity showed statistically different ORs by subtype for dose (*p* = 0.02) and duration (*p* < 0.01). Race-stratified: among black women, ever vs. never and especially long duration are more strongly associated with Luminal cancer [current vs. never OR 1.53 (1.04–2.26); duration > 20 yrs vs. never OR 2.06 (1.38–3.06)], with former smoking associated with increased basal-like risk [OR 1.71 (1.02–2.86)]. Among white women, associations are weaker or null for luminal; basal-like shows an inverse association with high dose [> 1 pack/day vs. never OR 0.38 (0.16–0.90)]. Overall pattern: long-term smoking is positively associated with luminal breast cancer, especially in black women, with no clear increase and some inverse signals for basal-like disease.
**Park et al., 2016 [23]**	USA; pooled data from 4 studies of African American women: Carolina Breast Cancer Study (CBCS), Women’s Circle of Health Study (WCHS), Black Women’s Health Study (BWHS), Multiethnic Cohort (MEC)	Pooled case-control analysis within the AMBER Consortium. CBCS & WCHS: population-based case-control; BWHS & MEC: prospective cohorts contributing nested case-control data. Diagnosis/enrolment periods: CBCS 1993–2014 (20–74 y); WCHS 2002–2013 (20–75 y); BWHS cohort initiated 1995 (21–69 y); MEC 1993–1996 (45–75 y).	African American women. Cases: first diagnosis of invasive breast cancer or DCIS with available smoking and receptor data. Controls: African American women without breast cancer, selected within each parent study (population controls in CBCS/WCHS; ~4 matched controls per case by birth year and questionnaire cycle in BWHS/MEC).	Eligible: 5,819 cases, 17,453 controls. After excluding 105 with missing smoking: 5,791 breast cancer cases and 17,376 controls. Subtype-specific: ER+ cases 3,099; ER− cases 1,511; triple-negative (TNBC) cases 694 (ER−/PR−/HER2−).	Smoking information was collected in each study via self-administered questionnaires or in-person interviews; all self-reported. Lifetime active smoking history up to index date (age at initiation, cessation, quantity). Variables were harmonised across studies for pooled analysis.	Harmonised active-smoking variables: (1) smoking status (never/former/current); (2) age at initiation (≤ 14, 15–17, 18–20, ≥ 21 y); (3) cigarettes/day (< 5, 5–14, 15–24, ≥ 25); (4) duration (< 10, 10–19, ≥ 20 years); (5) pack-years (< 10, 10–19, ≥ 20); (6) among parous women, years smoked before first birth (never smokers; smoked only after first birth; 1–5, 6–9, ≥ 10 years before first birth). Never-smokers are the reference. Passive smoking was also assessed, where available, but it is secondary.	Smoking history ascertained for the period prior to the index date: diagnosis date for cases; corresponding reference date for controls (matched by study-specific procedures). Analyses use smoking exposure up to that date only.	Pathology data from hospital records and/or cancer registries. Tumours classified by ER, PR, and HER2. Subtypes used in analysis: ER+ (any ER-positive), ER−, and TNBC defined as ER−/PR−/HER2−. HER2 is not available for some earlier cases; those with missing receptor data were excluded from subtype-specific models. Assays and cut-offs followed routine clinical practice in each centre; specific % thresholds were not detailed.	Multivariable unconditional logistic regression. Basic adjustment: age, study, calendar year of interview, geographic region. Fully adjusted model: additionally, education, age at menarche, age at first birth, parity, age at menopause, oral contraceptive use, estrogen-only therapy, combined estrogen + progestin therapy, BMI, family history of breast cancer, and alcohol use. All categorical with “unknown” levels as specified in the paper. Same adjustment set for the overall and subtype-specific models.	Overall breast cancer: in premenopausal women, both former and current smokers had modestly lower risk vs. never (OR ≈ 0.8, no dose-response by duration or pack-years). In postmenopausal women, long duration (≥ 20 y) and higher pack-years (≥ 20) were associated with modestly higher risk (OR ≈ 1.14–1.16). By subtype: for ER+ disease, long-term smoking (≥ 20 y) showed a small positive association (duration ≥ 20 y vs. never OR ≈ 1.11, *p*-trend ~0.03), while associations for ER− and TNBC were close to null (ORs around 1.0 for former/current, duration and pack-years, with no clear trends). Overall pattern: smoking has little to no association with ER− or TNBC, with only a modest increase in risk for long-term smoking in ER+ and postmenopausal African American women.
**Ellingjord-Dale et al., 2017 [13]**	Norway; nationwide Norwegian Breast Cancer Screening Program	Nested case-control within a population-based mammography screening cohort, 2006–2014. Women 50–69 invited every 2 years for two-view mammography; attendance ≈ 75%. Cases were identified through the Cancer Registry of Norway; for each case, 5 controls matched on year of birth (± 3 y) and year of last screening (± 3 y).	Women aged 50–69 years who attended screening 2006–2014, completed risk-factor questionnaires, and had no history of invasive cancer (except non-melanoma skin cancer) or DCIS before 1 Jan 2006. Cases: first invasive breast cancer (ICD-10 C50) with ER, PR, HER2 data. Controls: cancer-free, alive, resident in Norway at case diagnosis, matched as above.	Screening cohort: 344,348 women eligible. After excluding missing covariates, 4,952 breast cancer cases remained. For controls, after exclusions, 197,854 women; from these, 24,760 controls matched (5 per case). Subtype classification possible for 4,402 cases: luminal A-like 2,761; luminal B-like HER2− 709; luminal B-like HER2+ 367; HER2+ 204; triple-negative 361.	Self-administered questionnaires completed at the last screening before diagnosis for cases and corresponding screening round for controls (if missing, previous round used; ≈ 16.5% from earlier questionnaire). Smoking history self-reported.	Smoking status: never/past/current. Intensity: number of cigarettes per day (current): never, 1–4, 5–9, 10–19, ≥ 20. Cumulative exposure: pack-years = (avg cigarettes/day ÷ 20) × years smoked, categorised as < 2.5, 2.5–4.9, 5.0–9.9, 10.0–14.9, 15.0–19.9, ≥ 20. Smoking was also analysed at ages 30–39 and 40–49 years in supplementary tables, but the main subtype table uses current status, current intensity, and lifetime pack-years.	Exposures (including smoking) were taken from the last questionnaire before diagnosis (or prior round if missing), i.e., current status and intensity at/near the time of screening, always prior to diagnosis for cases and corresponding date for controls.	ER, PR, HER2 obtained from pathology reports submitted to the Cancer Registry. ER+: ≥ 10% nuclear staining 2006–Jan 2012; ≥ 1% from Feb 2012 onwards. PR+: ≥ 10% throughout. HER2: IHC 0/1+ = negative; 3+ = positive; 2+ confirmed by in situ hybridization. If IHC 2+ and ISH positive (or ISH positive with missing IHC) → HER2+; if IHC 2+ and ISH negative → HER2−. Subtypes (St Gallen-based): luminal A-like (ER+PR+HER2−); luminal B-like HER2− (ER+PR−HER2−); luminal B-like HER2+ (ER+PR±HER2+); HER2+ (ER−PR−HER2+); triple-negative (ER−PR−HER2−).	Conditional logistic regression matched on birth year & screening year. All ORs mutually adjusted for BMI (≤ 22, 23–25, 26–28, > 28 kg/m^2^), education (primary, high school, bachelor/master), age at menarche (9–12, 13, 14, 15–18 y), number of pregnancies ≥ 6 months (0, 1, 2, 3, ≥ 4), and menopausal status (pre, peri, post). For smoking models, additionally adjusted for alcohol intake (never, 1, 2, 3–4, ≥ 5 glasses/week) and physical activity (0, 1, 2–3, 4–5, ≥ 6 hours/week).	Overall breast cancer: smoking status: past vs. never OR 1.06 (95% CI 0.98–1.15); current vs. never OR 1.13 (1.03–1.23), *p*-trend = 0.006. Cigarettes/day (current): 10–19 vs. never OR 1.22 (1.06–1.39); ≥ 20 vs. never OR 1.41 (1.06–1.89), *p*-trend = 0.001. Pack-years: ≥ 20 vs. < 2.5 pack-years OR 1.26 (1.08–1.46), *p*-trend = 0.004. By subtype: for luminal A-like cancers, past vs. never OR 1.12 (1.01–1.23), current vs. never OR 1.18 (1.05–1.32), *p*-trend = 0.003. Current 10+ cigarettes/day vs. never OR 1.27 (1.07–1.50), and pack-years ≥ 20 vs. < 2.5 OR 1.27 (1.04–1.54), *p*-trend = 0.01. For luminal B-like HER2−, current smoking OR 1.22 (0.98–1.52); 10+ cigarettes/day vs. never OR 1.38 (1.00–1.89); pack-years ≥ 20 vs. < 2.5 OR 1.62 (1.09–2.40), *p*-trend = 0.03. For luminal B-like HER2+, HER2+ (ER−PR−HER2+) and triple-negative cancers, smoking status, intensity and pack-years showed no clear associations (ORs around 1.0, non-significant trends). Overall interpretation: smoking increases risk of luminal A-like and luminal B-like HER2− breast cancers, particularly at ≥ 10 cigarettes/day or ≥ 20 pack-years, with no detectable association for HER2+ or triple-negative disease.
**Gomes et al., 2022 [24]**	Brazil; state of Paraíba, Northeast Brazil; two breast-cancer reference centres (FAP, Campina Grande; HNL, João Pessoa) and public/rural primary care centres	Hospital-based case-control study. Cases: invasive operable breast cancer diagnosed and treated 2017–2020 at FAP or HNL. Controls: healthy women recruited in the same period from the same hospitals and three rural public health-care centres. Participants interviewed March 2017–March 2020; median time from diagnosis to interview ≈ 9 months.	Cases: women ≥ 18 years with invasive BC diagnosed ≤ 36 months before recruitment; no in situ tumours, BC recurrence, or previous other cancers. Controls: women without any cancer or chronic disease (e.g., diabetes, heart disease), age-matched to cases (± 5 years), only one control per family.	Total sample: 313 invasive BC cases and 321 controls. Of cases, 224 (71.6%) were postmenopausal. For molecular subtype analysis, 12 cases were excluded for missing IHC data, leaving 301 cases: luminal A 54 (17.9%), luminal B 175 (58.1%), HER2 29 (9.7%), TNBC 43 (14.3%).	Structured questionnaire administered face-to-face by study authors. Cases interviewed in chemotherapy/radiotherapy units; controls interviewed in waiting rooms of health-care centres. Information collected on: family history of cancer in first-degree relatives, alcohol consumption (ever/never and frequency), smoking (ever/never), oral contraceptive use (ever/never), age at menarche, parity, reproductive phase, etc. Height and weight for BMI from medical records (measured before treatment). Smoking, alcohol, and contraceptive use were all self-reported.	Main risk factors analysed: family history (yes/no); BMI categories (normal 18.5–24.99, overweight 25–29.99, obesity ≥ 30.0 kg/m^2^); alcohol consumption (ever/never, plus monthly frequency categories: never, 1–2, 3–7, ≥ 8 times/month); ever smoked (yes/no); oral contraceptive use (yes/no); menopausal status (pre/post); age at menarche (< 12 vs. ≥ 12 years); nulliparity (yes/no). For subtype models, these were entered into polytomous logistic regression with healthy controls as the reference.	Questionnaire responses and BMI measurements reflect pre-diagnosis lifetime history up to diagnosis/interview; cases were included if diagnosed within the previous 36 months (median 9 months before interview), so exposures predominantly pre-diagnostic but some potential for post-diagnosis change/recall bias.	ER, PR, and HER2 status were obtained from pathology reports. Subtypes are defined as: luminal A: ER+ and/or PR+, HER2−, Ki-67 < 14%; luminal B: ER+ and/or PR+ and HER2+, or ER+ and/or PR+, HER2−, Ki-67 ≥ 14%; HER2 subtype: HER2+, ER−, PR−; triple-negative (TNBC): ER−, PR−, HER2−. All tumours invasive.	Overall case-control models: logistic regression with backward selection. Final model adjusted for menopause status, age at menarche, smoking (ever/never), and age (categorical); exposures retained in the final model: family history, BMI, alcohol consumption, and contraceptive use. Subtype models: polytomous logistic regression with healthy controls as reference; final model adjusted for menopause status, nulliparity, smoking, age at menarche, and age (categorical).	Overall BC risk (all women, cases vs. controls, multivariable model): family history yes vs. no: OR 1.78 (95% CI 1.22–2.59). Obesity vs. normal BMI: OR 1.69 (1.08–2.63); overweight vs. normal: OR 1.37 (0.92–2.04). Alcohol consumption (ever vs. never): OR 2.21 (1.44–3.39). Contraceptive use (ever vs. never): OR 2.99 (2.09–4.28). Ever smoked (yes vs. no) was associated with BC in age-adjusted analysis (OR 1.51, 95% CI 1.07–2.12) but was not retained in the final multivariable model. Stratified by menopausal status: among postmenopausal women, obesity alone increased BC risk: OR 2.02 (1.22–3.37); alcohol consumption increased risk: OR 4.15 (2.13–8.11). Among premenopausal women, obesity was not associated; nulliparity increased BC risk: OR 4.19 (1.65–10.49), whereas among postmenopausal women nulliparity was protective (OR 0.36, 0.18–0.70). Alcohol dose-response: vs. never, 1–2 times/month OR 1.27 (0.71–2.27); 3–7 times/month OR 3.82 (1.96–7.43); ≥ 8 times/month OR 5.00 (2.00–12.51). Subtype-specific risks vs. controls (polytomous logistic regression, adjusted model): Family history increased risk of luminal A (OR 3.78, 1.90–7.52) and TNBC (OR 2.58, 1.27–5.23); association for luminal B attenuated to borderline (OR 1.49, 0.96–2.31). Obesity (vs. normal) increased risk mainly for TNBC (OR 4.06, 1.58–10.42) and luminal B (OR 1.87, 1.13–3.11); no clear effect for luminal A or HER2. Overweight showed non-significant positive trends for luminal A and TNBC. Alcohol consumption increased the risk of luminal A strongly: OR 7.08 (3.40–14.73), and luminal B more modestly: OR 1.77 (1.07–2.92); no clear association for HER2 or TNBC. Contraceptive use increased the risk of luminal A (OR 4.48, 2.09–9.58), luminal B (OR 3.08, 2.02–4.69), and HER2 (OR 4.89, 1.92–12.44), but not clearly TNBC (OR 1.57, 0.77–3.22). Early menarche (< 12 years) particularly increased TNBC risk (age-adjusted OR 4.54, 2.15–9.58). Overall pattern: obesity and alcohol are strongly linked to TNBC and luminal subtypes, respectively, while smoking shows only a modest crude association with overall BC and no clear independent subtype-specific effect after adjustment.
**Ihenacho et al., 2022 [25]**	USA; population-based in Los Angeles County (CA) and Metropolitan Detroit (Oakland, Wayne, Macomb counties), via SEER registries	Population-based case-control study of young-onset breast cancer (YOBC). Recruitment 2010–2015. Cases ascertained by rapid case ascertainment through LA and Detroit SEER; controls selected by area-based sampling from Census postal addresses, frequency-matched to cases on race (NHB/NHW), region, and 5-year age group.	US-born women, self-identified female, non-Hispanic Black (NHB) or non-Hispanic White (NHW), aged 20–49 years at reference date, residing in LA County or Metropolitan Detroit. Cases: incident, invasive, primary breast cancer diagnosed 2010–2015, confirmed histologically. Controls: cancer-free women meeting the same demographic and residence criteria.	In total 1,812 invasive YOBC cases (1,130 NHW, 682 NHB) and 1,381 controls (716 NHW, 665 NHB) completed interviews. Smoking status was missing for 18 (14 cases, 4 controls), leaving 1,798 cases and 1,377 controls for main smoking analyses. Tumour subtype data are missing for 130 cases, leaving 1,670 cases for subtype analyses. Subtypes: luminal A, luminal B, HER2-type, triple-negative (numbers not all given in text but all included in polytomous models).	In-person structured interview using a life-history calendar to enhance recall. Detailed lifetime personal cigarette smoking history was obtained: smoking status, age at start, periods of cessation, average cigarettes per day (CPD), total years smoked, timing of initiation relative to first full-term pregnancy (FFTP). Smoking is self-reported.	Ever smoking: ≥ 1 cigarette/day for ≥ 6 months (yes/no). Smoking status: never/formerly smoked /currently smoke; women who quit ≤ 1 year before reference date were classified as current smokers. CPD: < 5, 5–19, ≥ 20. Pack-years: (< 5, 5–19, ≥ 20), calculated as (CPD/20) × years smoked. Age at initiation: < 18, 18–24, ≥ 25 years. Time since initiation: < 20, 20–29, ≥ 30 years. Time since quitting among former smokers: 1–10, ≥ 10 years (vs. never). Timing relative to FFTP (parous women): initiated after FFTP vs. initiated before FFTP vs. never smoked. All smoking exposures use “never smoked” as reference.	Exposure history was defined up to the reference date: the date of invasive BC diagnosis for cases and the date 4 months before interview for controls. Lifetime smoking variables (status, CPD, pack-years, age at initiation, time since initiation, timing vs. FFTP) are constructed using data up to that reference date; thus pre-diagnostic for cases.	Tumour subtypes were derived from SEER pathology data (hospital/registry) based on ER, PR, HER2 and tumour grade. Categorised as: luminal A (ER/PR+, HER2−, grade 1/2); luminal B (ER/PR+, HER2+, any grade, or ER/PR+, HER2−, grade ≥ 3); HER2-type (ER−, PR−, HER2+); triple-negative (TNBC) (ER−, PR−, HER2−).	Multivariable logistic regression for overall YOBC and polytomous logistic regression for subtypes. All models sample-weighted. Adjustment set (final models): study site (LA/Detroit), age (20–29, 30–39, 40–49 years), household poverty level (HHP ≥ 200% vs. < 200% of federal poverty level), first-degree family history of BC (no/yes/unknown), BMI 12 months before reference date (underweight, normal, overweight, obese), lifetime cumulative alcohol intake (5 categories including abstainers), joint parity/age at FFTP (combined categories), and menopausal status (premenopausal vs. peri/post). Same covariates used for overall and subtype-specific models; BMI was also explored in sensitivity analyses (with and without adjustment, as potential mediator).	Overall YOBC (all subtypes combined): ever vs. never smoking was associated with increased YOBC risk: aOR 1.20 (95% CI 1.00–1.44). By subtype (ever vs. never): strong heterogeneity (*p* = 0.01). Increased risk for luminal A aOR 1.34 (1.06–1.68) and HER2-type aOR 1.97 (1.23–3.16); no association for luminal B aOR 1.04 (0.78–1.39) or TNBC aOR 0.92 (0.68–1.25). Smoking status: current vs. never significantly increased luminal A risk aOR 1.36 (1.02–1.81); former vs. never aOR 1.33 (0.98–1.71). For HER2-type, former vs. never aOR 2.41 (1.45–4.01), current vs. never aOR 1.58 (0.84–2.99). Dose/intensity: risk of HER2-type YOBC increased with higher CPD and pack-years; e.g., for HER2-type, higher categories of CPD and pack-years show monotonic rises in aORs. Age at initiation: ≥ 25 years vs. never was associated with increased YOBC overall aOR 1.91 (1.24–2.96), luminal A aOR 2.25 (1.32–3.84), and TNBC aOR 1.94 (1.03–3.64); while < 18 years vs. never increased HER2-type risk aOR 2.36 (1.36–4.09). Time since initiation: ≥ 30 years since initiation vs. never increased luminal A risk aOR 1.55 (1.07–2.26) and HER2-type aOR 2.77 (1.32–5.79). Timing vs. FFTP (parous): initiation before FFTP vs. never increased YOBC overall aOR 1.25 (1.02–1.54) and luminal A aOR 1.45 (1.11–1.89); HER2-type aOR 1.79 (0.99–3.25, not statistically significant). Little evidence of interaction by race or SEP; ever smoking increased overall YOBC, particularly among NHW women (aOR 1.39, 1.06–1.82) but not NHB (aOR 0.96, 0.70–1.31). Overall conclusion: lifetime smoking is positively associated with young-onset luminal A and HER2-type breast cancer, with no clear association for Luminal B or TNBC.
**Peñalver-Argüeso et al., 2023 [14]**	Spain; population-based multi-case-control study (MCC-Spain) in 12 provinces, with 22 collaborating hospitals for cases and population controls from general practitioner (GP) lists in the same catchment areas	Population-based case-control study. Recruitment 2008–2013. Incident, histologically confirmed invasive breast cancer cases identified soon after diagnosis from pathology/oncology services; controls randomly sampled from GP lists during the same period. No follow-up (point-in-time case-control).	Women aged 20–85 years, resident ≥ 6 months in the recruitment area, able to complete interview. Cases: incident invasive breast cancer, no previous breast cancer. Controls: cancer-free women from the same catchment population, with no history of breast cancer (or the specific tumour under study).	Total: 1,733 invasive breast cancer cases and 1,903 controls. Pathological subtype information available for 1,578 cases (91.1%): HR+ (ER or PR+, HER2−): 1,144; HER2+: 300; TN (ER−/PR−/HER2−): 134. Analyses stratified by menopausal status: pre/peri-menopausal 610 cases/547 controls; post-menopausal 1,122 cases/1,352 controls.	Standardised face-to-face interviewer-administered questionnaire. Detailed lifetime tobacco history plus sociodemographic, anthropometric (self-reported weight and height 1 year before interview), reproductive, family history and lifestyle variables. Women reporting < 100 cigarettes over their lifetime were classified as never smokers. All smoking information is self-reported.	Main smoking variables: smoking status 1 year before interview (never; former ≥ 10 years since quitting; former < 10 years; current); age at initiation (never, ≥ 18, < 18 years); duration (< 20, 20–30, > 30 years); intensity (< 15 vs. ≥ 15 cigarettes/day); pack-years (< 10, 10–25, > 25); among parous women, years smoking before first birth (< 10 or ≥ 10) and cigarettes/day before first birth (< 15 or ≥ 15). All contrasts use never smokers as reference.	All smoking variables refer to exposure up to 1 year before diagnosis (cases) or reference date (controls), to minimise reverse causation and allow a minimum latency.	ER, PR, HER2 data from hospital pathology/cancer registries. Subtypes grouped as: HR+: ER+ and/or PR+, without HER2 overexpression; HER2+: HER2 overexpressed, any ER/PR; TN: ER−, PR−, HER2−. Ki-67 not used. IHC cut-offs not explicitly detailed; classification reflects routine diagnostic practice.	Overall breast cancer models: unconditional logistic regression adjusted for age and province, then additionally for education, age at first birth, number of children, menopausal status, previous breast biopsies, family history of breast cancer and alcohol consumption. Pre/peri-menopausal models were further adjusted for oral contraceptive use; post-menopausal models were further adjusted for BMI, hormone replacement therapy and age at menopause. Subtype analyses: multinomial logistic regression (controls as reference) with the same covariate set (including menopausal/BMI/HRT terms).	All women (overall BC): smoking status 1 year before interview showed no clear association: vs. never, former ≥ 10 y OR ≈ 0.92; former < 10 y OR ≈ 0.87; current OR ≈ 1.02 (all CIs include 1; *p*-trend ≈ 0.97). Pre-/peri-menopausal: smoking tended to increase risk – starting smoking at ≥ 18 y OR ≈ 1.5; duration > 30 y OR ≈ 1.8; similar positive (mostly non-significant) trends for higher pack-years. Post-menopausal: smoking appeared protective, especially at low to moderate exposure and in overweight/obese women: duration > 30 y OR ≈ 0.69; intensity < 15 cig/day OR ≈ 0.70; pack-years < 10 OR ≈ 0.68; 10–25 OR ≈ 0.62 (no reduction at > 25 pack-years). In post-menopausal women, inverse associations were concentrated in BMI ≥ 25 kg/m^2^. By subtype: overall (all ages), no strong heterogeneity; in post-menopausal women, long-term former smoking (quit ≥ 10 y) was associated with reduced HER2+ risk (RRR ≈ 0.28) and low-intensity smoking with reduced TN risk (RRR ≈ 0.28), but numbers were small, and most other subtype-specific estimates were close to null.

BC: breast cancer; BMI: body mass index; CBCS: Carolina Breast Cancer Study; CI: confidence interval; CPD: cigarettes per day; CT: Clinical Trial; DCIS: ductal carcinoma in situ; DMV: Department of Motor Vehicles; ER: estrogen receptor; FAP: Fundação Assistencial da Paraíba; FFTP: first full-term pregnancy; FISH: fluorescence in situ hybridisation; GP: general practitioner; HER1: human epidermal growth factor receptor 1; HER2: human epidermal growth factor receptor 2; HNL: Hospital Napoleão Laureano; HR: hazard ratio; IHC: immunohistochemistry; ISH: in situ hybridisation; LA: Los Angeles; MEC: Multiethnic Cohort; MET-h/week: metabolic equivalent task hours per week; NC: North Carolina; NHB: Non-Hispanic Black; NHW: Non-Hispanic White; OR: odds ratio; OS: observational study; PR: progesterone receptor; PY: pack-years; SEER: Surveillance, Epidemiology, and End Results Program; TN: triple-negative; TNBC: triple-negative breast cancer; UNC: University of North Carolina; USA: United States of America; WHI: Women’s Health Initiative; YWHHS: Young Women’s Health History Study.

Several large cohort and population-based case-control studies have reported that ever smoking, current smoking, long duration, and higher cumulative exposure were associated with increased risk of ER-positive or luminal subtypes. Kabat et al. [22] found that former smoking and higher pack-year categories were linked to elevated risk of ER-positive breast cancer, with clear dose-response trends for cigarettes per day, duration, and cumulative exposure, whereas hazard ratios for TNBC remained close to unity and without dose-response. Kawai et al. [12] similarly showed increased risk of ER-positive disease among ever, current, and heavier smokers in young women, while odds ratios for TNBC were null across all smoking categories. In the Carolina Breast Cancer Study, Butler et al. [21] reported that long-term smoking (> 20 years) increased the risk of luminal tumors, particularly among Black women, whereas basal-like cancers showed no increase and, in some subgroups, even inverse associations at the highest dose levels.

Evidence from European cohorts was consistent with these findings. Ellingjord-Dale et al. [13] demonstrated that current smoking, higher intensity (≥ 10 cigarettes per day) and cumulative exposure (≥ 20 pack-years) were associated with increased odds of luminal A-like and luminal B-like HER2-negative tumors, with statistically significant dose-response trends. In contrast, HER2-positive and TNBC did not exhibit clear associations with smoking status, intensity, or pack-years. In the MCC-Spain study, Peñalver-Argüeso et al. [14] reported no substantial overall effect of smoking on breast cancer risk; however, stratified analyses suggested that any positive association was more apparent in pre/peri-menopausal women. Among postmenopausal women—particularly those overweight or obese—low-to-moderate cumulative smoking was associated with reduced risk for some subtypes, although these findings were based on relatively small numbers and should be interpreted with caution.

Studies restricted to specific populations also demonstrated broadly compatible results. Park et al. [23] (AMBER Consortium) found little or no overall association of smoking with breast cancer in African American women, yet long-term smoking was modestly associated with increased risk of ER-positive disease, while estimates for ER-negative and triple-negative tumors were close to null. Gomes et al. [24] reported that ever smoking was associated with breast cancer in age-adjusted analyses in a Brazilian population, but smoking did not remain an independent risk factor in adjusted models, and no clear subtype-specific pattern persisted. In the Young Women’s Health History Study, Ihenacho et al. [25] observed that ever and current smoking were associated with increased risk of luminal A-like tumors in women aged 20–49 years. Smoking also increased the risk of HER2-positive disease in this cohort, with higher cigarettes per day and higher pack-years particularly linked to HER2-positive cancers; by contrast, luminal B-like and triple-negative tumors did not show meaningful associations. Timing of smoking initiation appeared relevant as well: initiation before the first full-term pregnancy increased luminal A-like risk, while earlier initiation was associated with greater HER2-positive risk. Taken together, these studies suggest a possible, non-statistically significant association between active cigarette smoking and hormone receptor-positive or luminal breast cancer, particularly in relation to longer duration and greater cumulative exposure; however, the pooled estimate for current vs. never smoking did not reach statistical significance, so this finding should be interpreted cautiously.

### ER-positive breast cancer (current vs. never smoking)

**Figure 2:**
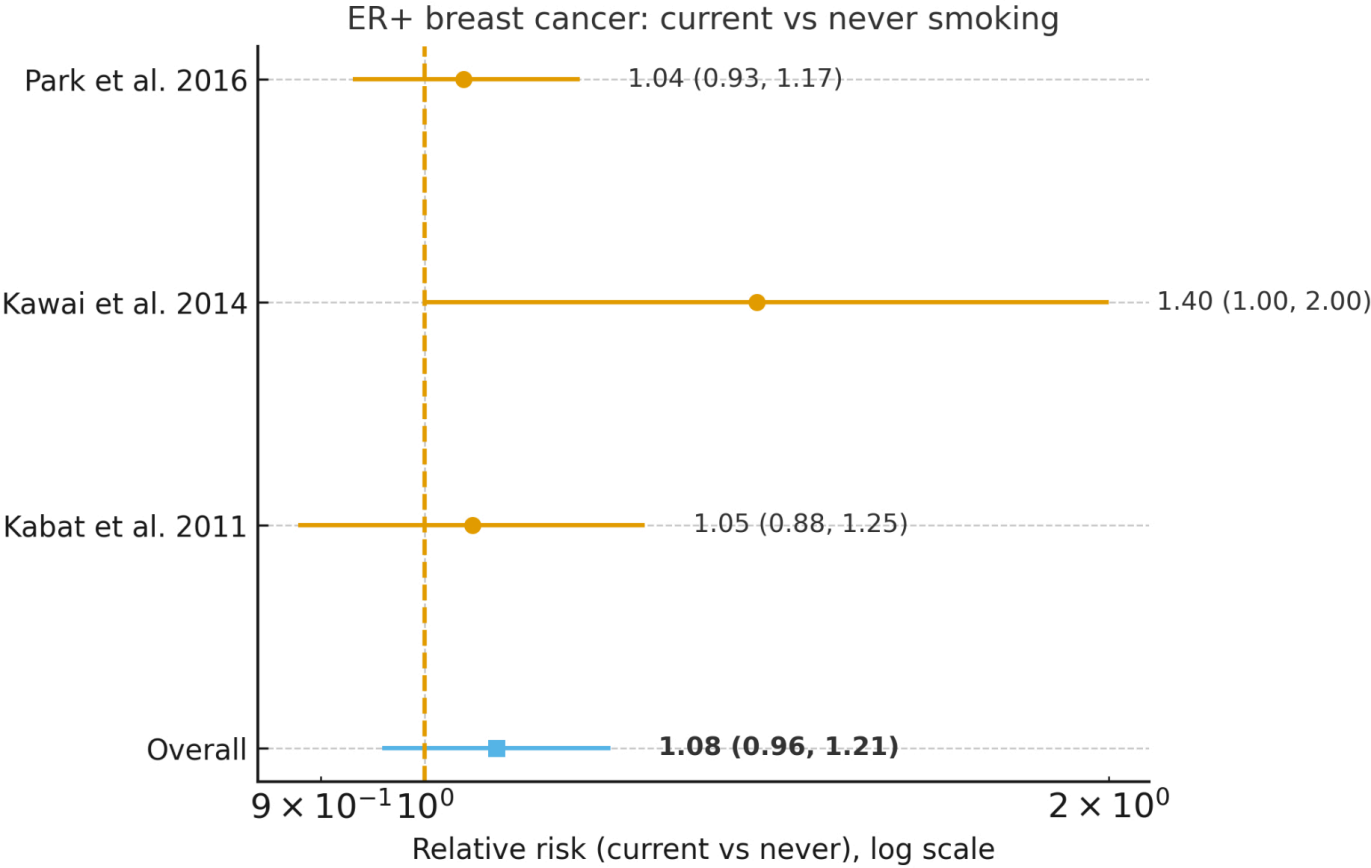
**Association between current cigarette smoking and risk of luminal (ER-positive) breast cancer (current vs. never smoking).** Forest plot showing relative risks (RRs) and 95% confidence intervals (CIs) from individual studies and the pooled random-effects estimate. The analysis shows a non-statistically significant association (pooled RR = 1.08, 95% CI 0.96–1.21). Statistical heterogeneity was low (*I*^2^ = 22.5%, *τ*^2^ = 0.0026). The horizontal axis is presented on a logarithmic scale, where RR = 1 represents the null value (no association).

In a random-effects meta-analysis of three studies [12, 22, 23], current smokers had a slightly higher, but not statistically significant, risk of ER-positive breast cancer compared with never smokers (pooled RR = 1.08, 95% CI 0.96–1.21, *p* = 0.22), with low heterogeneity (*I*^2^ = 22.5%, *τ*^2^ = 0.0026) (Figure 2).

### Triple-negative breast cancer (current vs. never smoking)

**Figure 3:**
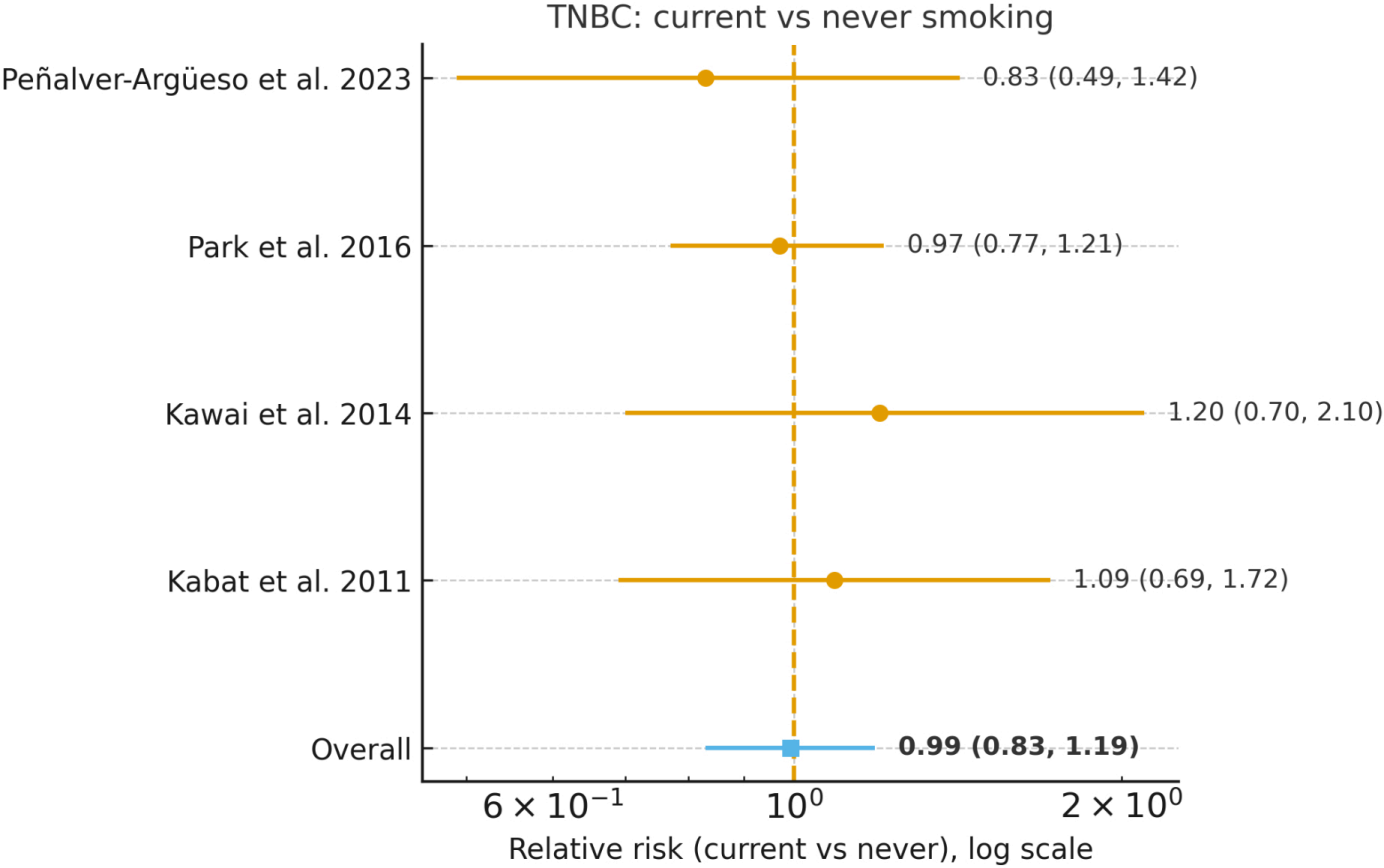
**Association between current cigarette smoking and risk of triple-negative breast cancer (TNBC) (current vs. never smoking).** Forest plot showing relative risks (RRs) and 95% confidence intervals (CIs) from individual studies and pooled random-effects estimate. No association was observed (pooled RR = 0.99, 95% CI 0.83–1.19). No between-study heterogeneity was detected (*I*^2^ = 0%, *τ*^2^ = 0.00). The horizontal axis is presented on a logarithmic scale, where RR = 1 represents the null value.

In a random-effects meta-analysis restricted to TNBC [12, 14, 22, 23], current smoking was not associated with TNBC risk. The pooled RR comparing current with never smokers was 0.99 (95% CI 0.83–1.19, *p* = 0.93), with no evidence of between-study heterogeneity (*I*^2^ = 0%, *τ*^2^ = 0.00). Across all four studies, the study-specific estimates clustered tightly around unity, indicating that active cigarette smoking does not appear to influence the incidence of TNBC (Figure 3).

### Triple-negative breast cancer (former vs. never smoking)

**Figure 4:**
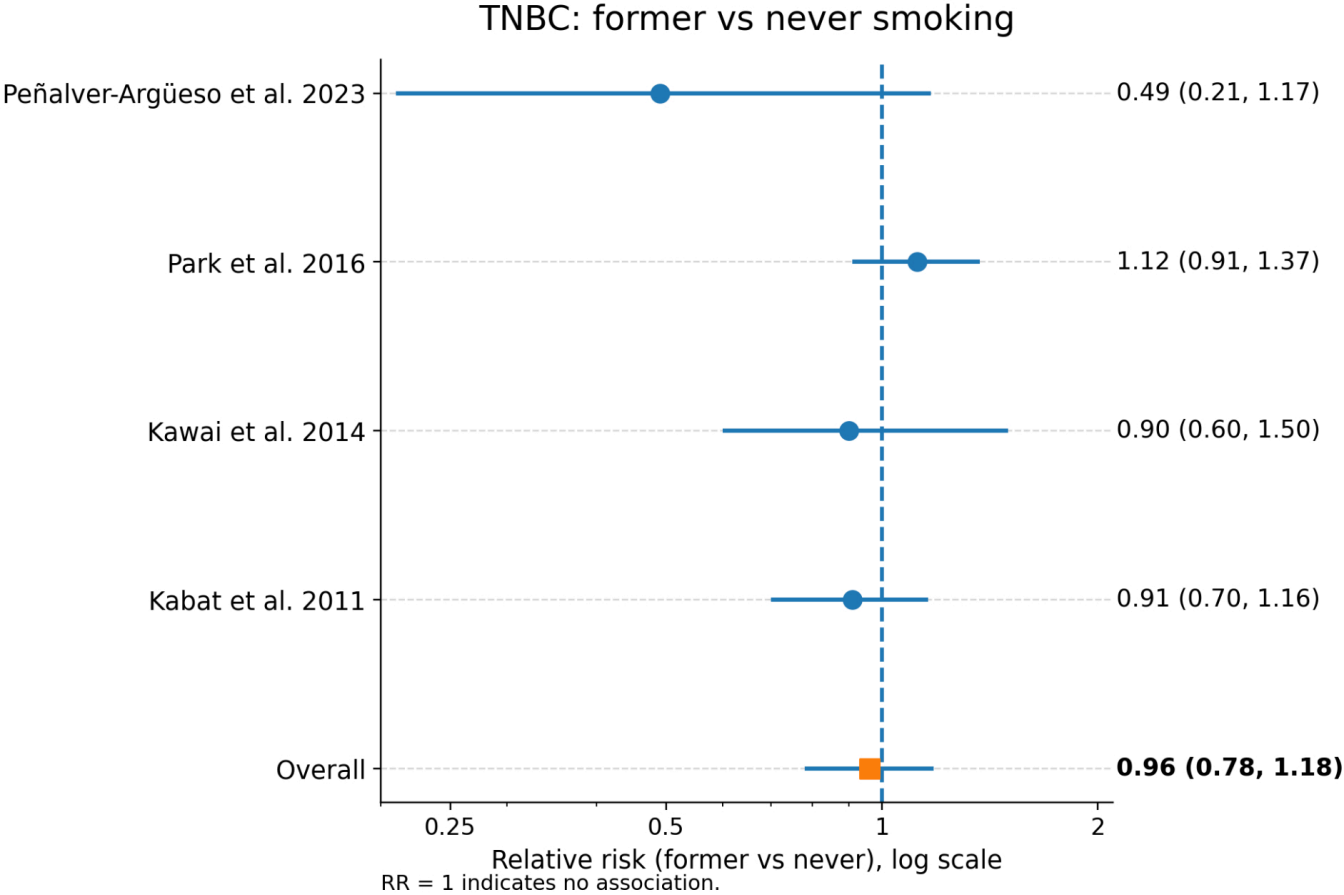
**Association between former cigarette smoking and risk of triple-negative breast cancer (TNBC) (former vs. never smoking).** Forest plot showing relative risks (RRs) and 95% confidence intervals (CIs) from individual studies and pooled random-effects estimate. No significant association was identified (pooled RR = 0.96, 95% CI 0.78–1.18). Heterogeneity was low to moderate (*I*^2^ = 34.3%, *τ*^2^ = 0.0149). The horizontal axis is presented on a logarithmic scale, where RR = 1 represents the null value.

In a random-effects meta-analysis of four studies [12, 14, 22, 23], former smoking was not associated with risk of TNBC. The pooled RR comparing former with never smokers was 0.96 (95% CI 0.78–1.18, *p* = 0.71), with low to moderate heterogeneity (*I*^2^ = 34.3%, *τ*^2^ = 0.0149). Study-specific estimates were centered around unity, indicating no clear evidence that cessation of smoking confers either increased or decreased risk of TNBC compared with never smoking (Figure 4).

### Associations between cigarette smoking and breast cancer molecular alterations

Four studies evaluated the relationship between cigarette smoking and molecular alterations in breast cancer, using diverse approaches including targeted DNA methylation analysis, receptor conversion between primary and recurrent tumors, immunohistochemistry-based molecular subtyping, and multi-omics profiling [15, 16, 17, 26]. Although heterogeneous in design and analytic strategies, these studies collectively suggest that smoking is associated with gene-specific epigenetic changes, receptor phenotype instability, greater genomic and immune disruption, and a shift toward more aggressive tumor subtypes (Table 2).

**Table 2:** Characteristics of studies assessing smoking in relation to tumor molecular profiles.

**First author, year**	**Country/Study population**	**Study design & sample (molecular subset)**	**Molecular data type (genes)**	**Platform/Assay**	**Bioinformatics/Statistical methods**	**Smoking exposure definition & analysis**	**Molecular outcomes/markers**	**Key findings on smoking-molecular associations**
Callahan et al., 2019 [26]	USA; women with incident primary breast cancer aged 35–79 years in the WEB (Western New York Exposures and Breast Cancer) study	Case-only analysis within population-based case—control study; FFPE tumor tissue from 718 breast cancer cases with methylation data (≈ 225 premenopausal, 493 postmenopausal; gene-specific *n* varies)	Tumor DNA methylation in promoter regions of 9 candidate genes: *SCGB3A1*, *CDKN2A*, *FHIT*, *GSTP1*, *SFN*, *BRCA1*, *RARB*, *CCND2*, *SYK*; methylation quantified as % and dichotomized as > median vs. ≤ median for each gene	Microdissected FFPE tumor DNA; bisulfite conversion (EZ DNA Methylation Kit, Zymo); targeted pyrosequencing (Qiagen pyrosequencing system) using commercial and custom primer sets; data processed with Pyro Q-CpG software	Gene-level methylation treated as binary outcome (above vs. ≤ median); unconditional logistic regression estimating ORs and 95% CIs for methylation by smoking exposures; models stratified by menopausal status and adjusted for age and ER status (plus pack-years for active smoking); cumulative SHS and pack-years dichotomized at median among exposed; period-specific exposures defined in 7 age windows; only cells with ≥ 5 subjects reported	Active smoking: detailed lifetime history by 7 age periods (< 21, 21–30, 31–40, 41–50, 51–60, 61–70, > 70 years); defined as ever smoking in each period, overall smoking status (never/former/current), and cumulative pack-years (total, dichotomized around median among exposed). SHS: among never smokers (< 100 cigarettes lifetime), residential and occupational SHS exposure in the same age windows; cumulative years of SHS summed across life and categorized (none, ≤ median, > median; e.g., ≤ 30 vs. > 30 years in postmenopausal women)	Gene-specific tumor promoter methylation status (high vs. low) for SCGB3A1, CDKN2A, FHIT, GSTP1, SFN, BRCA1, RARB, CCND2, SYK; no composite molecular signatures; ER/PR/HER2 and triple-negative status used as covariates, not primary outcomes	Premenopausal: active smoking before 21, 21–30, and 41–50 years associated with lower odds of SCGB3A1 hypermethylation (OR ≈ 0.25–0.30); smoking before 21 associated with higher GSTP1 methylation (OR ≈ 2.6); smoking at 31–40 associated with lower BRCA1 methylation (OR ≈ 0.09). Postmenopausal: active smoking at 41–50 strongly associated with higher FHIT methylation (OR ≈ 4.6) and at 51–60 with higher GSTP1 methylation (OR ≈ 2.3); current vs. never smokers had increased CDKN2A methylation (OR ≈ 2.1; *p*-trend ≈ 0.02); higher pack-years (> median) associated with increased CDKN2A methylation (OR ≈ 2.0). Among postmenopausal never-smokers, greater cumulative SHS was inversely associated with BRCA1 and SYK methylation (e.g., >30 years SHS vs. none for BRCA1 OR ≈ 0.3). No consistent associations for premenopausal SHS or for most other genes
Takada et al., 2020 [16]	Japan; women with resectable primary breast cancer undergoing curative surgery at Osaka City University Hospital (2007–2018); subset with biopsy/resection of recurrent lesions and known smoking history	Single-centre retrospective cohort of 989 primary breast cancer patients; recurrences in 77, of whom 50 (with paired primary-recurrent tissue and recorded smoking history) were included for molecular/smoking analyses; all were preoperative systemic-therapy-naïve	Protein expression of ER, PR, HER2 and Ki-67 in primary and recurrent tumors by immunohistochemistry; tumors classified into intrinsic subtypes: HRBC (ER and/or PR+), HER2BC (ER−/PR−/HER2+), TNBC (ER−/PR−/HER2−)	Standard immunohistochemistry on surgical and recurrent biopsy/resection specimens in institutional pathology lab; Ki-67 proliferation index evaluated with a 14% cutoff; imaging (US, CT, bone scintigraphy) used for staging but not for molecular classification	Concordance/discordance in receptor status (ER, PR, HER2) between primary and recurrent tumors evaluated; chi-square tests for associations between receptor conversion and clinicopathological factors; logistic regression to estimate ORs and 95% CIs for positive HER2 conversion by smoking status and pack-year categories; Kaplan–Meier curves and log-rank tests for progression-free survival (PFS) and post-recurrence survival (PRS); Cox proportional hazards models for univariate and multivariate prognostic analyses	Smoking history was recorded at the first visit (cigarettes/day and years of smoking); pack-years calculated as (cigarettes per day ÷ 20) × years; patients classified as smokers (any history) vs. non-smokers; 14/50 (28%) were smokers with median 30 pack-years (range 1.4–150); for HER2-conversion analyses, smokers were further grouped by pack-years (≤ 25, 25–50, > 50) vs. non-smokers; smoking assessed only up to surgery (no longitudinal updates)	Changes in IHC status of ER, PR, and HER2 between primary and recurrent tumors; intrinsic subtype change (HRBC/HER2BC/TNBC) at recurrence; observed conversion rates: ER negative conversion 3/50 (6%), ER positive conversion 1/50 (2%); PR negative conversion 15/50 (30%); HER2 positive conversion 6/50 (12%), no HER2 negative conversion; intrinsic subtype change in 5/50 (10%)	Positive HER2 conversion at recurrence was significantly more frequent in smokers (4/14; 28.6%) than in non-smokers (2/36; 5.6%) (*p* = 0.024); logistic regression showed smokers vs. non-smokers had higher odds of HER2 positive conversion (OR 6.8, 95% CI 1.082–42.731), with ORs increasing across higher pack-year categories (up to OR 17.0 for > 50 pack-years vs. non-smokers, albeit with wide CIs); smoking was not significantly associated with ER or PR conversion, intrinsic subtype change, or other clinicopathological variables
Wang et al., 2021 [17]	TCGA pan-cancer cohort (BLCA, CESC, ESCA, HNSC, KIRP, LUAD, LUSC); 2,317 tumor patients with recorded smoking history and multi-omics data	Retrospective multi-omics analysis of TCGA level-3 data across 7 smoking-related cancers; integrated RNA-seq, miRNA, DNA methylation, SNVs, CNVs, and clinical data (OS, DSS, PFI, stage, age, sex)	Multi-omics: mRNA expression (RNA-seq), miRNA expression, lncRNA expression, DNA methylation (Illumina HumanMethylation450), somatic SNVs, CNVs, immune/stromal scores, stemness indices; identification of 11 smoking-related methylation driver genes (*EIF5A2*, *GBP6*, *HGD*, *HS6ST1*, *ITGA5*, *NR2F2*, *PLS1*, *PPP1R18*, *PTHLH*, *SLC6A15*, *YEATS2*) and a 46-gene smoking-related prognostic signature; ceRNA network involving miRNAs (e.g., miR-193b-3p, miR-301b, miR-205-5p, miR-132-3p, miR-212-3p, miR-1271-5p, miR-137)	Public TCGA pipelines: RNA-seq [log_2_(TPM + 1)], Illumina 450K methylation, VarScan2 SNVs, masked CNV segments; CNVs summarized with GISTIC2.0; immune and stromal contexture from ssGSEA and ESTIMATE; chemotherapeutic response predicted using GDSC IC50 modeling (ridge regression via “pRRophetic”)	Survival differences by smoking history evaluated with Kaplan-Meier curves and Cox regression; multi-variable Cox models including smoking (non/former/current coded 0/1/2), age, sex, and stage; ssGSEA for 29 immune signatures; ESTIMATE for stromal/immune/estimate scores and tumor purity; BCR diversity, leukocyte fraction, neoantigens, HRD, CTA scores from published TCGA resources; stemness indices (mRNAsi, mDNAsi, DMPsi, ENHsi, EREG-mRNAsi, EREG-mDNAsi) from Malta et al.; mutation and CNV burden and landscapes analyzed with “maftools”; differential expression via edgeR; ceRNA network using miRcode, miRDB, TargetScan, miRTarBase; methylation driver genes defined by inverse correlation (*R* < −0.4, *p* < 0.05) between methylation and expression; 46-gene prognostic model built with univariate Cox + LASSO + multivariate Cox; ROC curves and C-index for model performance; nomograms with calibration for each cancer type	Smoking history derived from TCGA clinical data; patients categorized as non-smokers, former smokers, current smokers; in Cox models coded 0, 1, 2, respectively; no pack-years, intensity, or duration data; all analyses stratified/comparative across these three smoking-history groups (non vs. former vs. current) across tumor types	Multi-omics endpoints comparing non-, former-, and current smokers: 29 immune signatures; ESTIMATE immune/stromal/estimate scores and tumor purity; BCR richness/Shannon, leukocyte fraction, neoantigen load, intratumor heterogeneity, HRD and CTA scores; stemness indices; TMB; SNV and CNV landscapes and burdens; differentially expressed mRNAs/lncRNAs/miRNAs and ceRNA network; 11 DNA methylation driver genes and their expression; a 46-gene smoking-related risk score; predicted IC50 to multiple targeted and cytotoxic agents	Current smokers had the worst OS and DSS, former smokers intermediate, non-smokers best; smoking history was an independent prognostic factor for OS and DSS (current > former > never risk); former smokers showed highest immune cell infiltration and immune/ESTIMATE scores and lowest tumor purity; smokers (current and former) had higher BCR diversity, leukocyte fraction, neoantigen load, intratumor heterogeneity, HRD and CTA scores than non-smokers; smoking was associated with higher stemness indices (mRNAsi, mDNAsi, etc.), higher TMB, and increased SNV incidence in multiple genes (e.g., *TP53*, *TTN*, *MUC16*, *CSMD3*, *RYR2*, *LRP1B*, *USH2A*, *SYNE1*, *ZFHX4*, *FLG*, *XIRP2*, *PCLO*) and higher CNV gain/loss burden at key loci (e.g., 3q26, 8q24, 9p21 CDKN2A/B), with partial reduction but not complete reversal after cessation; smokers had higher predicted IC50 (reduced sensitivity) for many targeted and cytotoxic drugs, with non-smokers generally most sensitive and former smokers intermediate; ceRNA network highlighted several miRNAs as potential mediators of tobacco-related tumor biology; 11 methylation driver genes showed inverse methylation-expression relationships and were linked to smoking status; 46-gene model risk scores were highest in current smokers, intermediate in former smokers, lowest in non-smokers
Ferreira et al., 2024 [15]	Brazil; women with breast carcinoma treated in 2 public hospitals in São Paulo state	Longitudinal cohort of 208 women with breast cancer (age 25–65, all parous with ≥ 1 month breastfeeding); 80 smokers and 128 non-smokers; all had core biopsy with anatomopathology and immunohistochemistry, and were followed for 17 months	Immunohistochemistry-based molecular subtypes (gene expression surrogates): luminal A, luminal B, luminal hybrid, HER2 overexpression, triple-negative, and “others”	Standard IHC on histological sections with automated system: antigen retrieval in PTLink (Dako), incubation/development/counterstaining in AutoStainer Link; highly sensitive polymer detection and ready-to-use FLEX antibodies; molecular subtype assignment based on established IHC surrogate criteria from microarray gene-expression-defined subtypes	Descriptive statistics with Kolmogorov-Smirnov test for normality; continuous variables as mean ± SD; group comparisons by ANOVA; categorical variables by chi-square; odds ratio for severe vs. non-severe cancer (smokers vs. non-smokers, “neoadjuvant chemotherapy groups”) with 95% CI; Kaplan-Meier curves for survival by smoking status, log-rank test; *p* < 0.05 considered significant	Smoking is defined as regular use of ≥ 1 cigarette/day; 80 women were classified as smokers and 128 as non-smokers; no information on duration, intensity, or pack-years; smoking status assessed at baseline (diagnosis) and used as binary exposure (smoker vs. non-smoker) in all analyses	Tumor molecular subtype by IHC (luminal A, luminal B, luminal hybrid, HER2 overexpression, triple-negative); clinical stage (TNM, grouped as early 0–IIB vs. late III–IV); “severe cancer” operationalized via molecular profile and need for neoadjuvant chemotherapy; mortality during 17-month follow-up	Molecular profile distribution differed by smoking: among smokers, luminal A 24.0%, luminal B 31.3%, luminal hybrid 14.4%, HER2 overexpression 7.2%, triple-negative 19.0%, others 4.1%; among non-smokers, luminal A 35.9%, luminal B 35.9%, luminal hybrid 11.7%, HER2 overexpression 6.3%, triple-negative 10.1%, others 0.1%. Smokers had significantly lower luminal A (*p* = 0.035) and higher triple-negative frequency (*p* = 0.030). Triple-negative smokers were younger (mean 48.2 years) than triple-negative non-smokers (52.6 years, *p* = 0.005). Risk of more severe cancer (defined by neoadjuvant chemotherapy groups/molecular severity) was 5.5-fold higher in smokers than non-smokers (OR 5.5; 95% CI 3.0–10.0). Clinical stage distribution (I–IV) did not differ significantly between smokers and non-smokers

ANOVA: analysis of variance; BCR: B-cell receptor (repertoire); BLCA: bladder urothelial carcinoma; BRCA1: breast cancer 1, early-onset; CCND2: cyclin D2; CDKN2A: cyclin-dependent kinase inhibitor 2A; ceRNA: competing endogenous RNA; CESC: cervical squamous cell carcinoma and endocervical adenocarcinoma; CI: confidence interval; CNV: copy number variation; CSMD3: CUB and Sushi multiple domains 3; CTA: cancer-testis antigen; CT: computed tomography; DMPsi: DNA methylation-based stemness index; DNA: deoxyribonucleic acid; DSS: disease-specific survival; EIF5A2: eukaryotic translation initiation factor 5A2; ENHsi: enhancer-based stemness index; ER: estrogen receptor; EREG-mDNAsi: epigenetically regulated DNA methylation-based stemness index; EREG-mRNAsi: epigenetically regulated mRNA-based stemness index; ESCA: esophageal carcinoma; ESTIMATE: Estimation of STromal and Immune cells in MAlignant Tumours using Expression data; FFPE: formalin-fixed paraffin-embedded; FHIT: fragile histidine triad; FLG: filaggrin; GBP6: guanylate binding protein family member 6; GDSC: Genomics of Drug Sensitivity in Cancer; GSTP1: glutathione *S*-transferase Pi 1; HGD: homogentisate 1,2-dioxygenase; HER2: human epidermal growth factor receptor 2; HER2BC: HER2-positive breast cancer; HNSC: head and neck squamous cell carcinoma; HR: hazard ratio; HRBC: hormone receptor-positive breast cancer; HRD: homologous recombination deficiency; HS6ST1: heparan sulfate 6-*O*-sulfotransferase 1; IC50: half maximal inhibitory concentration; IHC: immunohistochemistry; ITGA5: integrin subunit alpha 5; Ki-67: Ki-67 proliferation index; KIRP: kidney renal papillary cell carcinoma; LASSO: least absolute shrinkage and selection operator; lncRNA: long non-coding RNA; LRP1B: low-density lipoprotein receptor-related protein 1B; LUAD: lung adenocarcinoma; LUSC: lung squamous cell carcinoma; mDNAsi: DNA methylation-based stemness index; miR: microRNA (prefix in miR IDs); miRNA: microRNA; mRNA: messenger RNA; mRNAsi: mRNA expression-based stemness index; MUC16: mucin 16, cell surface associated; NR2F2: nuclear receptor subfamily 2 group F member 2; OR: odds ratio; OS: overall survival; PCLO: piccolo presynaptic cytomatrix protein; PFI: progression-free interval; PFS: progression-free survival; PLS1: plastin 1; PPP1R18: Pprotein phosphatase 1 regulatory subunit 18; PR: progesterone receptor; PRS: post-recurrence survival; PTHLH: parathyroid hormone like hormone; RNA-seq: RNA sequencing; ROC: Receiver operating characteristic; RYR2: Ryanodine receptor 2; SCGB3A1: Secretoglobin family 3A member 1; SD: Standard deviation; SFN: Stratifin (14-3-3 sigma); SHS: second-hand smoke; SLC6A15: solute carrier family 6 member 15; SNV: single-nucleotide variant; ssGSEA: single-sample gene set enrichment analysis; SYK: spleen tyrosine kinase; SYNE1: spectrin repeat containing nuclear envelope protein 1; TCGA: The Cancer Genome Atlas; TMB: tumor mutational burden; TNBC: triple-negative breast cancer; TNM: tumor-node-metastasis staging system; TP53: tumor protein p53; TPM: transcripts per million; TTN: titin; US: ultrasonography; USH2A: usherin; WEB: Western New York Exposures and Breast Cancer study; XIRP2: xin actin-binding repeat-containing protein 2; YEATS2: YEATS domain containing 2; ZFHX4: zinc finger homeobox 4.

Callahan et al. [26] reported that smoking was linked to promoter methylation alterations in several cancer-related genes, with associations depending on both menopausal status and the timing of exposure. Among premenopausal women, smoking during adolescence and early adulthood was associated with decreased methylation of *SCGB3A1* and *BRCA1* but increased methylation of *GSTP1*. In contrast, postmenopausal smokers demonstrated higher methylation of *FHIT*, *GSTP1* and *CDKN2A*, with stronger effects among women with higher pack-year exposure. These findings indicate that tobacco exposure may induce epigenetic remodeling in breast tumors, affecting pathways related to genomic stability, cell-cycle control, and detoxification.

Takada et al. [16] found that smoking was strongly associated with HER2 receptor conversion from negative to positive between primary breast cancers and their recurrences. Smokers—particularly those with substantial cumulative exposure (> 50 pack-years)—had significantly higher odds of developing HER2-positive recurrence, whereas no meaningful associations were observed for ER or PR conversion. These results suggest a potential association between smoking and tumor phenotypic evolution, possibly contributing to treatment resistance and changes in therapeutic eligibility.

Wang et al. [17], analyzing multi-omic data across smoking-related cancers including breast cancer, demonstrated that smoking was associated with higher tumor mutational burden, increased copy-number alterations, and enrichment of mutations in several key cancer genes such as *TP53*, *MUC16*, and *TTN*. Smokers exhibited features of increased genomic instability, including higher homologous recombination deficiency, mutational burden, and altered immune profiles, suggesting a potential association with more aggressive and treatment-resistant tumor phenotypes.

Ferreira et al. [15] observed that smokers displayed a distinct shift in immunohistochemistry-defined molecular subtypes, with fewer luminal A-like tumors and a higher proportion of TNBC compared with non-smokers. Smokers also had a significantly higher likelihood of presenting with more severe tumor profiles requiring neoadjuvant chemotherapy. These findings reinforce the possibility that smoking is associated with more aggressive biological behavior at tumor presentation.

Taken together, these four studies demonstrate that smoking is associated not only with epigenetic and genomic alterations in breast tumors but also with phenotypic instability and a shift toward more aggressive molecular subtypes. Although the methodologies differ, the converging evidence suggests that cigarette smoking may be involved in breast cancer development and progression through multiple molecular alterations, including epigenetic changes, increased mutational burden, and receptor instability.

### Survival and recurrence outcomes

Across the prognostic and molecular cohorts, smoking at or around the time of diagnosis was more often associated with worse survival than with consistent differences in tumor biology, although the strength and presence of associations varied by study, subtype, and treatment (Table 3). In large population-based series, Loroña et al. [28] and Seibold et al. [29] showed that ever and especially current smoking were associated with higher risks of recurrence, breast cancer-specific mortality and all-cause mortality, with effect sizes often in the range of 30–100% excess risk and some evidence of stronger associations in triple-negative, HER2-positive and luminal A-like tumors, in NAT2 slow acetylators, and in women with higher pack-year exposure. Persson et al. [30] likewise reported approximately two-fold higher all-cause mortality in smokers overall and, importantly, markedly increased risks of breast cancer events, distant metastases, and death among older ER-positive women treated with aromatase inhibitors, whereas no clear adverse effect of smoking was seen in tamoxifen-treated patients, suggesting treatment-specific modification of prognosis. Ferreira et al. [15] observed substantially poorer short-term survival in smokers, with roughly doubled mortality over 17 months, and the pan-cancer TCGA analysis by Wang et al. [17] further supported a graded worsening of overall and disease-specific survival from never to former to current smokers, consistent with a broad detrimental impact of smoking on cancer outcomes. In contrast, several more selected or single-center studies did not identify smoking as an independent prognostic factor: Goldvaser et al. [18] found no association between smoking history or pack-years and disease-free, breast cancer-specific or overall survival in early ER-positive/HER2-negative patients evaluated with Oncotype DX; Schmidt et al. [31] reported almost identical disease-free and overall survival curves for smokers and non-smokers with TNBC; and Takada et al. [16] observed no significant differences in progression-free or post-recurrence survival between smokers and non-smokers despite a higher frequency of HER2 receptor conversion in smokers. Finally, Andres et al. [32] showed that smoking status alone was not strongly prognostic, but that integrating smoking history with tumor gene-expression profiles yielded smoking-specific multigene signatures with good discrimination for overall and disease-free survival, indicating that smoking may shape prognosis indirectly by interacting with particular molecular pathways rather than acting as a uniform risk factor across all tumors. Overall, these studies suggest that smoking is frequently, though not consistently, associated with worse survival and higher recurrence risk. This apparent discrepancy between large population-based cohorts and smaller subtype-restricted or single-center studies may be explained by several factors. Larger cohorts are more likely to capture the cumulative impact of smoking, including its interaction with comorbidities and competing risks, whereas smaller studies may be underpowered to detect modest prognostic effects. In addition, differences in treatment regimens (e.g., endocrine therapy vs. chemotherapy), variations in follow-up duration, and residual confounding by lifestyle and socioeconomic factors may contribute to inconsistent findings across studies. These factors may attenuate or obscure smoking-related effects in more selected or molecularly homogeneous populations.

**Table 3:** Molecular characteristics and survival outcomes in studies assessing smoking history and breast cancer prognosis.

**First author, year**	**Country/Study population**	**Study design & sample (molecular subset)**	**Molecular data type (genes)**	**Platform/Assay**	**Bioinformatics/Statistical methods**	**Smoking exposure definition & analysis**	**Molecular outcomes/markers**	**Key findings on smoking-molecular associations**	**Survival/Recurrence outcomes**
Andres et al., 2015 [32]	USA; women with primary invasive breast carcinoma whose frozen tumors were archived in the University of Louisville breast cancer biorepository (diagnosed 1988–1996)	Retrospective prognostic study using tumor specimens with long-term follow-up; gene discovery in 247 LCM-procured carcinoma samples (microarray, 22,000 genes) of which 165 had known smoking status; validation set of 98 tumors analysed by RT-qPCR (48 cigarette smokers, 50 never-smokers) with complete outcome data	Tumor mRNA expression (RT-qPCR) of 23 candidate genes: *NAT1*, *NAT2*, *COMT*, *SOD1*, *SOD2*, *BRCA1*, *BRCA2*, *CYP1A1*, *APOC1*, *ARID1B*, *CTNNBL1*, *MSX1*, *UBE2F*, *IRF2*, *NCOA1*, *LECT2*, *THAP4*, *RIPK1*, *AGPAT1*, *C7orf23*, *CENPN*, *CETN1*, *YTHDC2*; candidates selected from microarray contrasts of smokers vs. non-smokers and recurrent vs. disease-free cases plus literature-based smoking/breast-cancer genes	Frozen tumor sections (median 60% carcinoma cells) → RNA isolation (RNeasy), quality check (Agilent Bioanalyzer), reverse transcription (Superscript III); SYBR Green RT-qPCR with gene-specific primers (Primer Express), ACTB as reference and Universal Human Reference RNA as calibrator; expression quantified as −ΔΔCt (log2-scale)	Univariate Cox models for OS and DFS for each gene and clinical covariate; BH correction for multiple testing. For prognostic modelling, data repeatedly split (1,000×) into 70% training/30% test sets separately for smokers and never-smokers; LASSO-penalized Cox used for variable selection; permutation tests to define significance thresholds for gene selection; multivariable Cox models (gene-only, genes + clinical covariates, clinical-only) fitted; L2 penalty added when needed for convergence; predictive performance assessed by C-index and Kaplan-Meier curves for high- vs. low-risk groups; additional models in combined cohort used interaction terms between smoking status and gene expression to test effect modification	Smoking history was obtained from clinical records. In the discovery microarray cohort, 66 women were confirmed cigarette smokers with pack-year data, and 99 were never-smokers. In the qPCR cohort, women were classified as cigarette smokers versus never-smokers; analyses were stratified by smoking status rather than dose. No information on secondhand smoke exposure was reported.	Continuous tumor expression of individual genes (−ΔΔCt; evaluated per two-fold increase) for 23 candidates; multigene prognostic signatures (6–8 genes) for OS and DFS in smokers and never-smokers; focus on genes repeatedly selected in LASSO models: in smokers, *CYP1A1*, *CETN1*, *NCOA1*, *IRF2*, *CENPN*, *LECT2*, *NAT1*, *RIPK1*; in never-smokers, *IRF2*, *CYP1A1*, *CETN1*, *NAT2*; interaction terms for smoking × *CYP1A1*, *LECT2*, *CETN1* in combined models	In smokers, higher expression of **CYP1A1** was strongly associated with lower mortality (HR per doubling 0.66, 95% CI 0.51–0.85) and lower recurrence risk (HR 0.77, 95% CI 0.66–0.90); higher **CENPN** expression was associated with increased mortality (HR 1.71, 95% CI 1.15–2.54) and recurrence (HR 1.37, 95% CI 1.03–1.84); **IRF2** showed a protective effect for OS (HR 0.78, 95% CI 0.62–0.98). In never-smokers, **IRF2** remained protective for OS (HR 0.81, 95% CI 0.69–0.95) and DFS (HR 0.86, 95% CI 0.75–0.99), while CETN1 showed a weaker, borderline adverse association with DFS. Smoking-gene interaction analyses in the combined cohort identified significant interactions for smoking status with **CYP1A1, LECT2, and CETN1**, indicating that the prognostic effect of these genes differs between smokers and never-smokers. Multigene signatures (≈ 7–8 genes) in smokers achieved high predictive accuracy (median C-index ≈ 0.8 for OS and ≈ 0.73 for DFS), whereas analogous signatures in never-smokers had only modest discrimination (C-index ≈ 0.59)	Outcomes: overall survival (time to death) and disease-free survival (time to first recurrence or death). Smoking status **alone** was not significantly associated with OS or DFS (HR for smokers vs. never-smokers ≈ 0.8–0.95), but integrating smoking history with tumor gene expression substantially improved prognostic stratification among smokers; gene-based models outperformed Adjuvant! Online risk scores in smokers (C-index ~0.79 vs. 0.69 for OS; ~0.75 vs. 0.68 for DFS), while in never-smokers gene-based and Adjuvant Online models performed similarly (C-indices ~0.59–0.62)
Goldvaser et al., 2017 [18]	Israel; women with early-stage ER-positive, HER2-negative breast cancer treated at Davidoff Cancer Center, Rabin Medical Center, whose tumors were sent for Oncotype DX testing (diagnosed 4/2005–3/2012)	Retrospective single-centre cohort of 662 women (median age 61 years; 78% postmenopausal). All had ER+/HER2− early breast cancer (stage I–IIIA) with available pathology and Oncotype DX results	Multigene expression-based recurrence score: Oncotype DX 21-gene RT-PCR assay (reported as continuous RS and categorized low 0–17, intermediate 18–30, high ≥ 31). Conventional IHC markers: ER, PR, HER2, Ki-67, p53; histologic subtype and grade	Oncotype DX is performed on FFPE tumor tissue (central RT-PCR assay; ER/PR/HER2 measured by RT-PCR as part of ODX). Local pathology: IHC for ER/PR using modified H-score (0–3), Ki-67 (% positive nuclei), HER2 by IHC with reflex FISH for 2+ cases per ASCO guidelines; standard histopathology for angiolymphatic and perineural invasion	Continuous variables: mean ± SD, compared by *t*-tests; categorical variables: *χ*² or Fisher’s exact tests. OS: Kaplan-Meier and log-rank. DFS and breast cancer–specific survival (BCSS): Cox proportional hazards models with Fine & Gray competing-risk correction (competing events: non-cancer death for BCSS, death without recurrence for DFS). Multivariable models adjusted for age, menopausal status, ethnicity, tumor size, nodal status and grade	Smoking data obtained at first oncology visit. Current smokers: actively smoking at diagnosis. Ever smokers: any history of smoking; never smokers: no history. Pack-years were recorded and used to define heavy smokers (≥ 30 pack-years) vs. < 30. Groups were compared as: ever vs. never; current vs. former + never; ≥ 30 vs. < 30 pack-years. No data on secondhand smoke, age at initiation, or pack-years before first pregnancy; smoking status not updated during follow-up	Oncotype DX RS (median 17; range 0–88; 50.6% low, 38.3% intermediate, 11.1% high). Tumor size, nodal status, stage, histologic type (IDC, ILC, other), grade, ER/PR IHC intensity, Ki-67 (%), p53 (%), angiolymphatic and perineural invasion	Smoking history, status, and pack-years were not associated with tumor size, nodal involvement, stage, histologic type, grade, ER/PR intensity, Ki-67, p53, or Oncotype DX RS (mean RS similar across all smoking groups). The only significant differences were higher angiolymphatic invasion (10.4% vs. 5.1%, *p* = 0.045) and perineural invasion (8.3% vs. 3.5%, *p* = 0.031) in current smokers vs. others; no clear pack-year gradient. Authors concluded that smoking had no clinically significant influence on tumor burden, Oncotype RS, or most pathological characteristics in this ER+/HER2− cohort	Overall 5-year DFS 95.7%, BCSS 98.5%, OS 98.5%. Recurrence rates: 3.6% (low RS), 5.3% (intermediate), 11% (high; *p* = 0.036); high RS was associated with worse DFS (HR 0.40 for low vs. high RS, 95% CI 0.16–0.98). Smoking variables were not associated with DFS, BCSS, or OS: ever vs. never smokers—DFS HR 0.73 (95% CI 0.32–1.68), BCSS HR 0.53 (0.12–2.38), OS HR 0.80 (0.22–2.92); heavy vs. lighter smokers—DFS HR 0.85 (0.26–2.80), BCSS HR 0.73 (0.09–5.70), OS HR 1.50 (0.33–6.78). Multivariable analyses adjusted for clinicopathologic factors yielded similar non-significant results, indicating no detectable impact of smoking on prognosis in this subgroup
Loroña et al., 2024 [28]	USA; women 20–69 years with first primary invasive breast cancer diagnosed 2004–2015 in Seattle-Puget Sound SEER region (King, Pierce, Snohomish counties); oversampling of TN and HER2-overexpressing tumors	Population-based prospective cohort of 3,876 cases (2,153 luminal ER+, 1,252 TN, 471 HER2-overexpressing); outcomes: any and site-specific recurrence, breast cancer-specific mortality, all-cause mortality; recurrence subset *n* = 3,197 (excludes stage IV, very-early recurrences, and those without medical record review)	Clinical molecular subtypes by ER/PR/HER2: luminal (ER+; luminal A and B combined), triple-negative (ER−/PR−/HER2−), HER2-overexpressing (ER−/HER2+); no genomic or epigenomic profiling	ER, PR, HER2 status abstracted from clinical pathology; HER2 determined by IHC with reflex FISH for equivocal cases; subtype assignment based on joint ER/PR/HER2 status per standard criteria	Multivariable Cox proportional hazards models for recurrence, breast cancer-specific mortality and all-cause mortality, fitted overall and separately by subtype; models mutually adjusted for alcohol and smoking and for age, year of diagnosis, stage, hypertension, diabetes, race/ethnicity and BMI; subtype used as stratification variable in overall analyses; cause-specific Cox models for site-specific recurrences (locoregional, distant, lung, liver, bone, brain); inverse probability weighting to account for under-sampling of luminal cases; multiple imputation by chained equations for covariates; tests for trend across pack-year categories and for interaction with alcohol; time-dependent coefficient models to compare early (< 5 y) vs. late (≥ 5 y) hazards	Smoking at or just before diagnosis from structured interviewer questionnaires, supplemented by medical records when needed; status categorized as never, former, current smoker; “ever smoker” = former + current. Pack-years (total lifetime) were calculated and analysed among ever smokers in categories (< 10 vs. ≥ 10 pack-years; trend test) and as dose-response within subtypes; primary exposure in the main models was smoking status at diagnosis; limited post-diagnostic data used only for concordance checks (91% same status pre/post)	Molecularly defined subtypes (luminal, TN, HER2-overexpressing); any recurrence and site-specific recurrence (locoregional, distant, bone, lung, liver, brain); breast cancer-specific death; all-cause death; also timing of recurrence and death by subtype (e.g., luminal cases more likely to recur ≥ 5 years after diagnosis)	Among TN cases, ever smoking vs. never was associated with a higher risk of any recurrence (HR 1.33, 95% CI 1.01–1.74) and current smoking with an even higher risk (HR 1.59, 95% CI 1.07–2.35). Current smoking was associated with increased distant recurrence overall (HR 1.53, 95% CI 1.02–2.30). Ever and current smoking were associated with > 50–80% higher risk of recurrence to bone among luminal cases and overall (cause-specific models). Pack-years ≥ 10 tended to show higher recurrence and mortality risks than < 10 pack-years, particularly for breast cancer-specific and all-cause mortality, although trends were sometimes imprecise. No clear effect modification by menopausal status or year of diagnosis; no joint effect between alcohol and smoking.	Ever smoking was associated with ~30–50% higher breast cancer-specific mortality across all cases, luminal, and TN subtypes and ~53–61% higher all-cause mortality across subtypes, with larger HRs for ≥ 10 pack-years. Current smoking at diagnosis was associated with 50–131% higher breast cancer-specific mortality and 122–158% higher all-cause mortality across subtypes. Associations were similar over time, with somewhat stronger effects of current smoking on early (< 5 y) breast cancer death in luminal tumors and late (≥ 5 y) death in TN tumors. Overall, smoking history at diagnosis was a consistent adverse prognostic factor; no survival benefit observed in any subtype.
Persson et al., 2016 [30]	Sweden; women with first primary breast cancer operated at Skåne University Hospital, Lund (2002–2012)	Population-based prospective cohort of 1,116 women; 51 with preoperative therapy excluded → 1,065 analysed for patient/tumour characteristics; 1,016 with invasive non-metastatic tumours included in survival analyses; 891 had ER+ tumours (endocrine-treatment subset)	Routine clinical markers: ER, PR, HER2 (no Ki-67 routinely); used to define ER+ endocrine-treated cohorts; no genomic or epigenomic profiling	Pathology at regional hospital lab: ER and PR by IHC (positive if >10% nuclei stained, per Swedish clinical practice); HER2 amplification assessed from 2005 onwards in patients <70 years with invasive tumours; standard histopathology for size, grade, nodal status	Patient/Tumour characteristics compared by smoking with *χ*²/Fisher’s exact tests and Mann-Whitney *U*; Kaplan-Meier curves and log-rank tests for breast cancer events, distant metastasis, and all-cause death; Cox proportional hazards models to estimate adjusted HRs (95% CIs) overall and within treatment strata; adjustment for tumour size ≥ 21 mm or muscle/skin involvement, any nodal involvement, grade III, ER status, age (continuous), BMI ≥ 25 kg/m^2^, and, in extended models, receipt of radiotherapy, chemotherapy, tamoxifen, and aromatase inhibitors; stratified analyses by endocrine treatment type (tamoxifen vs. AIs) and age (< 50 vs. ≥ 50 years)	Smoking status self-reported preoperatively via questionnaire (self-defined non-smoker, smoker, or occasional smoker; plus cigarette categories for last week: 0, 1–5, 6–10, 11–15, 16–20, ≥ 20); “Smokers” = those identifying as smokers/occasional smokers or reporting any cigarettes in the prior week; 223/1,065 (21%) were smokers at baseline; follow-up questionnaires at 3–6 months and 1 year showed < 1% of preoperative non-smokers started smoking and ~10% of smokers quit, indicating stable status; analyses use preoperative smoking (yes/no); no pack-years or former-smoking history, and former smokers grouped with never smokers	ER, PR, HER2 status and standard pathological features; primary outcomes: “breast cancer events” (local/regional recurrence, new breast cancer, or distant metastasis), distant metastasis alone, and death from any cause; endocrine-response analyses restricted to ER+ tumours, with subgroups by endocrine therapy: tamoxifen ever, AI ever, neither; additional stratification by age (< 50 vs. ≥ 50 years) and by receipt of chemotherapy or radiotherapy	Smokers were younger, leaner, had smaller breast volume, fewer children and younger age at first full-term pregnancy, and were more often hormone receptor-negative (lower ER+ and PR+ frequencies) than non-smokers. Overall, preoperative smoking showed no significant association with risk of breast cancer events (adjHR 1.45, 95% CI 0.95–2.20), but was linked to ~two-fold higher all-cause mortality (adjHR 2.03, 95% CI 1.29–3.21). In ER+ patients ≥ 50 years treated with aromatase inhibitors (AIs) (*n* = 309), smoking was strongly associated with worse outcomes: breast cancer events (adjHR 2.97, 95% CI 1.44–6.13), distant metastases (adjHR 4.19, 95% CI 1.81–9.72), and death (adjHR 3.52, 95% CI 1.59–7.81); absolute event rates were 17.5 vs. 48.2 per 1,000 person-years in non-smokers vs. smokers. Among ≥ 50-year ER+ patients treated with tamoxifen (TAM) (*n* = 408), smoking was not significantly associated with breast cancer events (adjHR 1.58, 95% CI 0.76–3.30). In chemotherapy-treated and in radiotherapy-only groups overall, smoking did not materially affect events, though a weak association in radiotherapy-treated patients disappeared after excluding AI users; within radiotherapy + AI-treated subgroup, smokers had ~four-fold higher event risk (adjHR 4.13, 95% CI 1.66–10.26). After excluding all AI-treated patients, smoking was not associated with breast cancer events or distant metastasis, but showed a borderline increased all-cause mortality (adjHR 1.82, 95% CI 1.01–3.26). Authors conclude that smoking may specifically impair AI effectiveness, whereas it does not appear to influence response to tamoxifen	Outcomes: breast cancer events (122 events; 76 distant metastases) and 97 deaths over median 5.1 years’ follow-up; overall, smokers had about double the risk of all-cause death vs. non-smokers, but no clear increase in breast cancer events. In ER+ ≥ 50-year AI-treated patients, smoking was associated with markedly shorter event-free, metastasis-free and overall survival (adjHRs ~3–4), while in TAM-treated patients survival did not differ by smoking status. Study suggests smoking is an adverse prognostic factor particularly in AI-treated women, and may need to be considered when choosing endocrine therapy
Schmidt et al., 2020 [31]	Germany; women with TxNxM0 triple-negative breast cancer (TNBC) treated at the Department of Gynecology, Obstetrics and Reproductive Medicine, University Medical School of Saarland (2004–2018)	Retrospective single-centre chart review of 197 TNBC patients; all ER−/PR−/HER2− at diagnosis; 84 received neoadjuvant chemotherapy (NACT), 87 adjuvant chemotherapy, 26 no chemotherapy; median follow-up 41.4 months	No genomic/epigenomic profiling; TNBC defined by lack of ER, PR, HER2 expression in routine pathology	Standard institutional pathology; ER/PR/HER2 IHC used only to establish TNBC status (all triple negative); no additional molecular assays reported	Descriptive statistics for baseline characteristics; OS and DFS analysed by Kaplan-Meier curves; group comparisons by log-rank test with two-sided *α* = 0.05; no multivariable Cox models; secondary analyses of OS/DFS by weight change (> 3 kg) and parity (> 3 pregnancies), and of pathologic complete response (pCR) rates by BMI, smoking, alcohol, physical activity and parity	Smoking habit was recorded as yes/no at baseline; smokers further described as regular (*n* = 35) or occasional (*n* = 12), total 47/197 (23.9%); no pack-years, intensity, age at initiation, or former-smoker category; exposure used as binary variable (smoker vs. non-smoker) in Kaplan-Meier/log-rank analyses of OS and DFS; no information on secondhand smoke	Clinical TNBC only (no additional markers); primary endpoints: overall survival (OS) and disease-free survival (DFS) by BMI, smoking, alcohol, physical activity and parity; secondary outcome: pCR (ypT0 ypN0/pN0) after NACT by these lifestyle factors; also explored impact of weight change > 3 kg and > 3 pregnancies on OS/DFS	Smoking habit did not influence OS or DFS. Log-rank *p*-values for smokers vs. non-smokers: OS *p* = 0.9892, DFS *p* = 0.6040 (Kaplan-Meier curves B1/B2 on page 4). Similarly, BMI, alcohol, physical activity and parity showed no significant association with OS or DFS (e.g., OS by BMI *p* = 0.4720; DFS *p* = 0.2272; alcohol OS *p* = 0.6515, DFS *p* = 0.7460). None of these lifestyle factors, including smoking, affected the probability of achieving pCR after NACT (34/84, 40.38% overall), nor did weight change > 3 kg or > 3 pregnancies impact outcomes. Authors conclude that in this TNBC cohort, smoking and other lifestyle factors studied were not prognostic for disease course.	During follow-up, 34/197 (17.3%) had recurrence, 51 (25.9%) developed metastases, and 51 (25.9%) died. Neither OS nor DFS differed significantly by smoking status or other lifestyle factors; survival curves by smoking and alcohol on page 4 show almost overlapping trajectories. Thus, no evidence that smoking at diagnosis alters recurrence risk or survival in women with TNBC in this series.
Seibold et al., 2014 [29]	Germany; postmenopausal women aged 50–74 years with invasive breast cancer in the population-based MARIE/MARIEplus study (Hamburg and Rhein-Neckar–Karlsruhe regions), diagnosed 2001–2005	Prospective cohort; 3,340 women with invasive breast cancer (after excluding in situ and prior non-breast malignancy) for mortality analyses; 2,857 with stages I–IIIA and clear staging (excludes NACT, stage IIIB–IV, early events) for recurrence analyses; NAT2 genotyped in 2,399	Clinicopathologic subtypes based on ER, PR, HER2, and grade: luminal A-like (ER/PR+, HER2−, grade 1–2), luminal B-like (ER/PR+, any HER2, grade 4), HER2+ non-luminal (ER−/PR−/HER2+), triple-negative (ER−/PR−/HER2−). Germline NAT2 genotype (slow vs. fast acetylator) from 5 polymorphisms (rs1041983, rs1799929, rs1799930, rs1208, rs17999312)	ER/PR status and grade from routine histopathology; HER2 by IHC ± confirmatory testing per local practice; NAT2 genotyping on blood DNA using Sequenom MassARRAY (iPLEX GOLD / hME Assay); NAT2 alleles classified into rapid (*4, *12A/B, *13A) vs. non-rapid (slow) acetylators	Delayed-entry multivariable Cox regression (PHREG, SAS) with age at diagnosis and region as strata; endpoints: all-cause, breast cancer-specific, non-breast-cancer mortality, and recurrence (local/regional/contralateral/distant). Models adjusted for tumour size, nodal status, metastasis, grade, joint ER/PR status, BMI, alcohol, mode of detection, radiotherapy, HRT at diagnosis, CVD, diabetes; proportional hazards checked with martingale residuals. Effect modification was tested by stratified Cox and interaction terms for NAT2 status, BMI (< 25 vs. ≥ 25 kg/m^2^), alcohol (< 12 vs. ≥ 12 g/day), and molecular subtype	Smoking before diagnosis from standardized face-to-face interview. Ever smokers: ≥ 100 cigarettes lifetime. Current smokers: smoked within the year before diagnosis; former smokers: previously smoked but not in last year; never smokers: < 100 cigarettes lifetime. Pack-years = packs/day × years; categories: never, < 10, 10–20, ≥ 20 pack-years. Cigarettes/day: < 10 vs. ≥ 10. Time since cessation in former smokers: < 10, 10–20, ≥ 20 years. Main exposure for effect-modification analyses: current vs. never/former combined	Molecular/Clinical markers: ER/PR/HER2–/grade-based subtypes (luminal A-like, luminal B-like, HER2+ non-luminal, triple-negative); NAT2 slow vs. fast acetylator status; standard TNM; outcomes: all-cause, breast cancer-specific, non-breast-cancer mortality, and any recurrence	Overall, current vs. never/former smoking was associated with higher all-cause mortality (HR 1.39, 95% CI 1.10–1.76) and non-breast-cancer mortality (HR 1.96, 95% CI 1.28–2.99), with non-significant trends for breast cancer-specific mortality (HR 1.23, 95% CI 0.93–1.64) and recurrence (HR 1.16, 95% CI 0.86–1.57). Risk of non-breast-cancer death increased with dose (per 5 pack-years HR 1.12, 95% CI 1.07–1.18; per 5 cigarettes/day HR 1.22, 95% CI 1.11–1.34). Effect-modification analyses showed substantially stronger smoking effects in NAT2 slow acetylators: current vs. never/former HRs for slow vs. fast acetylators were 1.93 vs. 1.28 (all-cause), 1.77 vs. 1.09 (breast cancer-specific), and 2.76 vs. 1.83 (non-breast-cancer mortality), though heterogeneity tests were underpowered. By subtype, current smoking doubled all-cause mortality in luminal A-like (HR 2.08, 95% CI 1.40–3.10) and triple-negative tumours (HR 1.93, 95% CI 1.02–3.65), and markedly increased recurrence risk in HER2+ non-luminal tumours (HR 3.64, 95% CI 1.22–10.8); no clear associations were seen in luminal B-like tumours. For non-breast-cancer mortality, risks were strongly elevated only in normal-weight women (BMI < 25 kg/m^2^: HR 2.52, 95% CI 1.52–4.15; BMI ≥ 25: HR 0.94, 95% CI 0.38–2.36; Phet = 0.04), and particularly in women consuming ≥ 12 g/day of alcohol (HR 3.38, 95% CI 1.32–8.69). Authors conclude smoking is an adverse prognostic factor, especially in NAT2 slow acetylators and in luminal A-like, HER2+ and triple-negative subtypes	Over median 5.7 years’ follow-up, 449 deaths (323 breast cancer-related) and 322 recurrences occurred. Current smoking increased all-cause mortality and non-breast-cancer mortality overall, with strongest absolute and relative excess in NAT2 slow acetylators and in certain molecular subtypes (luminal A-like, triple-negative, HER2+). Associations for breast cancer-specific mortality and recurrence were weaker overall but became more evident in these subgroups. Findings support targeted emphasis on smoking cessation in breast cancer patients, particularly those with NAT2 slow acetylator status or with luminal A-like, HER2+ or triple-negative tumours.
Ferreira et al., 2024 [15]	Brazil; women with breast carcinoma treated in 2 public hospitals in São Paulo state	Longitudinal cohort of 208 women with breast cancer (age 25–65, all parous with ≥ 1 month breastfeeding); 80 smokers and 128 non-smokers; all had core biopsy with anatomopathology and immunohistochemistry, and were followed for 17 months	Immunohistochemistry-based molecular subtypes (gene expression surrogates): luminal A, luminal B, luminal hybrid, HER2 overexpression, triple-negative, and “others”	Standard IHC on histological sections with automated system: antigen retrieval in PTLink (Dako), incubation/development/counterstaining in AutoStainer Link; highly sensitive polymer detection and ready-to-use FLEX antibodies; molecular subtype assignment based on established IHC surrogate criteria from microarray gene-expression-defined subtypes	Descriptive statistics with Kolmogorov-Smirnov test for normality; continuous variables as mean ± SD; group comparisons by ANOVA; categorical variables by chi-square; odds ratio for severe vs. non-severe cancer (smokers vs. non-smokers, “neoadjuvant chemotherapy groups”) with 95% CI; Kaplan-Meier curves for survival by smoking status, log-rank test; *p* < 0.05 considered significant	Smoking was defined as regular use of ≥ 1 cigarette/day; 80 women classified as smokers and 128 as non-smokers; no information on duration, intensity, or pack-years; smoking status assessed at baseline (diagnosis) and used as binary exposure (smoker vs. non-smoker) in all analyses	Tumor molecular subtype by IHC (luminal A, luminal B, luminal hybrid, HER2 overexpression, triple-negative); clinical stage (TNM, grouped as early 0–IIB vs. late III–IV); “severe cancer” operationalized via molecular profile and need for neoadjuvant chemotherapy; mortality during 17-month follow-up	Molecular profile distribution differed by smoking: among smokers, luminal A 24.0%, luminal B 31.3%, luminal hybrid 14.4%, HER2 overexpression 7.2%, triple-negative 19.0%, others 4.1%; among non-smokers, luminal A 35.9%, luminal B 35.9%, luminal hybrid 11.7%, HER2 overexpression 6.3%, triple-negative 10.1%, others 0.1%. Smokers had significantly lower luminal A (*p* = 0.035) and higher triple-negative frequency (*p* = 0.030). Triple-negative smokers were younger (mean 48.2 years) than triple-negative non-smokers (52.6 years, *p* = 0.005). Risk of more severe cancer (defined by neoadjuvant chemotherapy groups/molecular severity) was 5.5-fold higher in smokers than non-smokers (OR 5.5; 95% CI 3.0–10.0). Clinical stage distribution (I–IV) did not differ significantly between smokers and non-smokers	Over 17 months, mortality was 39.5% in smokers vs. 20% in non-smokers; Kaplan-Meier analysis showed significantly lower survival among smokers (log-rank *p* = 0.01), with an estimated risk of death 2.2 times higher in smokers (95% CI 1.19–4.58); mean survival time for non-smokers was ~240 days; no multivariable survival modeling reported beyond smoking status
Takada et al., 2020 [16]	Japan; women with resectable primary breast cancer undergoing curative surgery at Osaka City University Hospital (2007–2018); subset with biopsy/resection of recurrent lesions and known smoking history	Single-centre retrospective cohort of 989 primary breast cancer patients; recurrences in 77, of whom 50 (with paired primary–recurrent tissue and recorded smoking history) were included for molecular/smoking analyses; all were preoperative systemic-therapy-naïve	Protein expression of ER, PR, HER2 and Ki-67 in primary and recurrent tumors by immunohistochemistry; tumors classified into intrinsic subtypes: HRBC (ER and/or PR+), HER2BC (ER−/PR−/HER2+), TNBC (ER−/PR−/HER2−)	Standard immunohistochemistry on surgical and recurrent biopsy/resection specimens in institutional pathology lab; Ki-67 proliferation index evaluated with a 14% cutoff; imaging (US, CT, bone scintigraphy) used for staging but not for molecular classification	Concordance/Discordance in receptor status (ER, PR, HER2) between primary and recurrent tumors evaluated; chi-square tests for associations between receptor conversion and clinicopathological factors; logistic regression to estimate ORs and 95% CIs for positive HER2 conversion by smoking status and pack-year categories; Kaplan-Meier curves and log-rank tests for progression-free survival (PFS) and post-recurrence survival (PRS); Cox proportional hazards models for univariate and multivariate prognostic analyses	Smoking history was recorded at the first visit (cigarettes/day and years of smoking); pack-years were calculated as (cigarettes per day ÷ 20) × years; patients classified as smokers (any history) vs. non-smokers; 14/50 (28%) were smokers with median 30 pack-years (range 1.4–150); for HER2-conversion analyses, smokers were further grouped by pack-years (≤ 25, 25–50, > 50) vs. non-smokers; smoking assessed only up to surgery (no longitudinal updates)	Changes in IHC status of ER, PR, and HER2 between primary and recurrent tumors; intrinsic subtype change (HRBC/HER2BC/TNBC) at recurrence; observed conversion rates: ER negative conversion 3/50 (6%), ER positive conversion 1/50 (2%); PR negative conversion 15/50 (30%); HER2 positive conversion 6/50 (12%), no HER2 negative conversion; intrinsic subtype change in 5/50 (10%)	Positive HER2 conversion at recurrence was significantly more frequent in smokers (4/14; 28.6%) than in non-smokers (2/36; 5.6%) (*p* = 0.024); logistic regression showed smokers vs. non-smokers had higher odds of HER2 positive conversion (OR 6.8, 95% CI 1.082–42.731), with ORs increasing across higher pack-year categories (up to OR 17.0 for > 50 pack-years vs. non-smokers, albeit with wide CIs); smoking was not significantly associated with ER or PR conversion, intrinsic subtype change, or other clinicopathological variables	PFS (from recurrence to progression or death) and PRS (from recurrence to death) were defined and analysed; median postoperative follow-up for the 50 recurrent cases was 2,128 days; no significant difference in PFS (*p* = 0.102, log-rank) or PRS (*p* = 0.140, log-rank) between smokers and non-smokers; in univariate Cox models, worse PFS was associated with adjuvant chemotherapy after surgery (HR 3.734, 95% CI 1.316–10.115) and intrinsic subtype change at recurrence (HR 3.889, 95% CI 1.083–11.236), and worse PRS with biopsied distant metastasis (HR 8.527, 95% CI 1.114–52.010), but smoking history was not an independent prognostic factor in multivariate analyses
Wang et al., 2021 [17]	TCGA pan-cancer cohort (BLCA, CESC, ESCA, HNSC, KIRP, LUAD, LUSC); 2,317 tumor patients with recorded smoking history and multi-omics data	Retrospective multi-omics analysis of TCGA level-3 data across 7 smoking-related cancers; integrated RNA-seq, miRNA, DNA methylation, SNVs, CNVs and clinical data (OS, DSS, PFI, stage, age, sex)	Multi-omics: mRNA expression (RNA-seq), miRNA expression, lncRNA expression, DNA methylation (Illumina HumanMethylation450), somatic SNVs, CNVs, immune/stromal scores, stemness indices; identification of 11 smoking-related methylation driver genes (*EIF5A2*, *GBP6*, *HGD*, *HS6ST1*, *ITGA5*, *NR2F2*, *PLS1*, *PPP1R18*, *PTHLH*, *SLC6A15*, *YEATS2*) and a 46-gene smoking-related prognostic signature; ceRNA network involving miRNAs (e.g., miR-193b-3p, miR-301b, miR-205-5p, miR-132-3p, miR-212-3p, miR-1271-5p, miR-137)	Public TCGA pipelines: RNA-seq [log_2_(TPM + 1)], Illumina 450K methylation, VarScan2 SNVs, masked CNV segments; CNVs summarized with GISTIC2.0; immune and stromal contexture from ssGSEA and ESTIMATE; chemotherapeutic response predicted using GDSC IC50 modeling (ridge regression via “pRRophetic”)	Survival differences by smoking history evaluated with Kaplan-Meier curves and Cox regression; multi-variable Cox models including smoking (non/former/current coded 0/1/2), age, sex, and stage; ssGSEA for 29 immune signatures; ESTIMATE for stromal/immune/estimate scores and tumor purity; BCR diversity, leukocyte fraction, neoantigens, HRD, CTA scores from published TCGA resources; stemness indices (mRNAsi, mDNAsi, DMPsi, ENHsi, EREG-mRNAsi, EREG-mDNAsi) from Malta et al.; mutation and CNV burden and landscapes analyzed with “maftools”; differential expression via edgeR; ceRNA network using miRcode, miRDB, TargetScan, miRTarBase; methylation driver genes defined by inverse correlation (R < −0.4, *p* < 0.05) between methylation and expression; 46-gene prognostic model built with univariate Cox + LASSO + multivariate Cox; ROC curves and C-index for model performance; nomograms with calibration for each cancer type	Smoking history derived from TCGA clinical data; patients categorized as non-smokers, former smokers, and current smokers; in Cox models coded as 0, 1, 2, respectively; no pack-years, intensity or duration data; all analyses stratified/comparative across these three smoking-history groups (non vs. former vs. current) across tumor types	Multi-omics endpoints comparing non-, former-, and current smokers: 29 immune signatures; ESTIMATE immune/stromal/estimate scores and tumor purity; BCR richness/Shannon, leukocyte fraction, neoantigen load, intratumor heterogeneity, HRD and CTA scores; stemness indices; TMB; SNV and CNV landscapes and burdens; differentially expressed mRNAs/lncRNAs/miRNAs and ceRNA network; 11 DNA methylation driver genes and their expression; a 46-gene smoking-related risk score; predicted IC50 to multiple targeted and cytotoxic agents	Current smokers had the worst OS and DSS, former smokers intermediate, non-smokers best; smoking history was an independent prognostic factor for OS and DSS (current > former > never risk); former smokers showed highest immune cell infiltration and immune/ESTIMATE scores and lowest tumor purity; smokers (current and former) had higher BCR diversity, leukocyte fraction, neoantigen load, intratumor heterogeneity, HRD and CTA scores than non-smokers; smoking was associated with higher stemness indices (mRNAsi, mDNAsi, etc.), higher TMB, and increased SNV incidence in multiple genes (e.g., *TP53*, *TTN*, *MUC16*, *CSMD3*, *RYR2*, *LRP1B*, *USH2A*, *SYNE1*, *ZFHX4*, *FLG*, *XIRP2*, *PCLO*) and higher CNV gain/loss burden at key loci (e.g., 3q26, 8q24, 9p21 CDKN2A/B), with partial reduction but not complete reversal after cessation; smokers had higher predicted IC50 (reduced sensitivity) for many targeted and cytotoxic drugs, with non-smokers generally most sensitive and former smokers intermediate; ceRNA network highlighted several miRNAs as potential mediators of tobacco-related tumor biology; 11 methylation driver genes showed inverse methylation-expression relationships and were linked to smoking status; 46-gene model risk scores were highest in current smokers, intermediate in former smokers, lowest in non-smokers	OS and DSS significantly differed by smoking group with graded worsening from never to former to current smokers; no significant overall difference in PFI between smoking groups, although 10–15-year PFI tended to be best in non-smokers; smoking history remained an independent predictor of OS and DSS in multivariate Cox models; the 46-gene risk score was an independent risk factor for OS across cancer types and showed good predictive accuracy for 1-, 3-, 5-year OS (and also DSS and PFI), with nomograms (risk score + clinical variables) achieving good calibration and C-indices across tumor types

ACTB: beta-actin; AGPAT1: 1-acylglycerol-3-phosphate *O*-acyltransferase 1; AI: aromatase inhibitor; AIs: aromatase inhibitors; APOC1: apolipoprotein C1; ARID1B: AT-rich interaction domain-containing protein 1B; ASCO: American Society of Clinical Oncology; BCSS: breast cancer-specific survival; BH: Benjamini-Hochberg (multiple-testing correction); BRCA2: breast cancer 2, early-onset; C7orf23: chromosome 7 open reading frame 23; CENPN: centromere protein N; CETN1: centrin 1; COMT: catechol-*O*-methyltransferase; CTNNBL1: catenin beta-like 1; CVD: cardiovascular disease; DFS: disease-free survival; ER+: estrogen receptor-positive; FISH: fluorescence in situ hybridisation; HRT: hormone replacement therapy; IDC: invasive ductal carcinoma; ILC: invasive lobular carcinoma; IRF2: interferon regulatory factor 2; LCM: laser-capture microdissection; LECT2: leukocyte cell-derived chemotaxin 2; MARIE: population-based German breast cancer study (MARIE study); MARIEplus: Extended MARIE cohort; MSX1: Msh homeobox 1; NAT1: *N*-acetyltransferase 1; NAT2: *N*-acetyltransferase 2; NACT: neoadjuvant chemotherapy; NCOA1: nuclear receptor coactivator 1; Oncotype DX: 21-gene RT-PCR recurrence score assay; p53: tumor protein p53; pCR: pathological complete response; RNeasy: RNeasy RNA extraction kit; RIPK1: receptor-interacting serine/threonine-protein kinase 1; RS: recurrence score; RT: reverse transcription; RT-qPCR: reverse transcription quantitative polymerase chain reaction; RT-PCR: reverse transcription polymerase chain reaction; SOD1: superoxide dismutase 1; SOD2: superoxide dismutase 2; SYBR: SYBR Green fluorescent dye; TAM: tamoxifen; THAP4: THAP domain-containing protein 4; UBE2F: ubiquitin-conjugating enzyme E2 F; USA: United States of America.

## Discussion

The findings suggest, at most, a modest and non-statistically significant trend toward increased risk of hormone receptor-positive or luminal breast cancer. Therefore, any potential association should be interpreted cautiously and not considered conclusive. In contrast, the limited but consistent molecular data suggest that smoking is linked to specific epigenetic and genomic alterations, receptor phenotype instability, and a shift towards more aggressive biological features, while prognostic studies generally, though not uniformly, show worse outcomes in smokers compared with non-smokers.

The overall pattern observed in this review is broadly consistent with large contemporary meta-analyses. Macacu et al. [8] reported pooled RRs of 1.12 for current and 1.09 for ever smokers compared with never smokers, with stronger associations among women who initiated smoking before first childbirth. Similarly, a recent dose-response meta-analysis identified small but statistically significant increases in risk across smoking categories, with linear relationships for both intensity and duration [10]. Gaudet et al. [11] also reported an approximately 10–15% higher risk of invasive breast cancer overall, with stronger associations for ER-positive disease and weaker, often non-significant findings for ER-negative tumors. In this context, our findings suggest, at most, a modest and non-statistically significant tendency toward increased risk in hormone receptor-positive or luminal tumors, which should be interpreted cautiously.

The adverse prognostic impact of smoking observed in this review is consistent with prior studies. Current smoking at diagnosis has been associated with increased breast cancer-specific and overall mortality, whereas former smoking shows weaker or no associations [33, 34]. Subsequent analyses have reported approximately 30% higher breast cancer-specific mortality among smokers [35], with consistent confirmation in later meta-analyses [36]. Large pooled data further indicate hazard ratios of 1.28 for breast cancer-specific mortality and 1.52 for all-cause mortality among current smokers [37], with more recent studies supporting dose-response relationships with cumulative exposure [38]. These findings support smoking as an adverse prognostic factor, although the magnitude of effect may vary by subtype and treatment context.

Overall, this review refines the existing literature by integrating epidemiological, molecular, and prognostic evidence. While epidemiological data suggest a possible, but non-definitive, association with luminal subtypes, molecular and clinical findings indicate that smoking may be linked to more aggressive tumor characteristics and poorer outcomes in certain patient groups. This supports the hypothesis that smoking may act more as a modifier of tumor biology and progression rather than a strong determinant of breast cancer incidence.

Biologically, these findings are plausible. Tobacco smoke contains carcinogens that promote DNA damage, oxidative stress, inflammation, and epigenetic alterations [7, 14–17]. Smoking-associated changes in promoter methylation of key genes, including FHIT, GSTP1, CDKN2A, and BRCA1, further support a role in genomic instability and tumor progression [15, 26]. In parallel, smoking-related alterations in mutational burden, homologous recombination deficiency, and immune microenvironment may contribute to more aggressive tumor behavior [17].

The apparent lack of association with TNBC incidence, contrasted with evidence of more aggressive phenotypes and receptor conversion in smokers [15, 16], suggests that smoking may not substantially influence tumor initiation in this subtype but may instead promote tumor evolution and phenotypic instability. Alternatively, heterogeneity in subtype classification and exposure assessment may obscure true associations.

From a clinical perspective, these findings support the inclusion of breast cancer among smoking-related harms, although the associated risk appears modest and should be communicated accordingly [8–11, 12–14, 21–25]. In patients with established disease, the consistent association between smoking and poorer outcomes, along with adverse molecular features [15–17, 28–30], underscores the importance of systematic smoking assessment and cessation support in oncology care. Although current evidence does not justify treatment stratification based on smoking status, smoking may represent a modifiable factor contributing to prognosis.

Finally, smoking-related molecular alterations overlap with pathways targeted by existing therapies, suggesting potential future relevance for risk stratification and treatment selection. For example, tumors with higher mutational burden or homologous recombination deficiency may have differential responses to immunotherapy or PARP inhibitors, while epigenetic changes may be therapeutically reversible [17, 26, 32]. These hypotheses remain to be validated but highlight the importance of integrating lifestyle exposures into precision oncology research.

### Strengths and limitations

This review has several strengths. First, it specifically focuses on subtype-defined and molecularly characterized breast cancer, an aspect that has been under-represented in previous summaries of smoking and breast cancer, which mostly addressed overall incidence and passive smoking [8–10]. Second, we applied a comprehensive and pre-registered search strategy across multiple databases, supplemented by grey literature and citation tracking, with explicit inclusion criteria and duplicate screening and data extraction, thereby reducing the risk of selection bias. Third, we used random-effects models and restricted pooling to studies with reasonably comparable exposure and outcome definitions, and we assessed risk of bias using an established tool adapted for observational and translational designs.

However, important limitations must be acknowledged. The number of studies eligible for meta-analysis was limited, particularly for specific subtype-exposure combinations, resulting in imprecise pooled estimates and restricted ability to explore heterogeneity or conduct subgroup analyses. Subtype definitions varied across studies, with some using simple ER/PR/HER2 immunohistochemistry and others applying extended IHC panels or gene-expression-based classifiers. These approaches are not fully interchangeable, particularly for luminal B-like, HER2-enriched, and basal-like categories, and classification differences may have contributed to the inconsistent results for HER2-positive disease and to the difficulty in detecting subtle subtype-specific effects.

Exposure assessment also differed substantially, spanning ever/never status, current/former categorization, intensity, duration, pack-years, and timing of initiation relative to first full-term pregnancy, with varying degrees of detail and accuracy. All studies relied on self-reported smoking, which is subject to misclassification. Residual confounding by alcohol intake, adiposity, reproductive factors, and socioeconomic status is likely, especially in observational studies that did not repeatedly update smoking exposure or comprehensively measure correlated lifestyle variables. For prognostic analyses, immortal time bias, changes in smoking behavior after diagnosis, and competing risks from other smoking-related diseases may further complicate interpretation. In addition, reliance on self-reported smoking exposure and the limited availability of detailed metrics such as duration, pack-years, and changes in smoking behavior over time may have introduced nondifferential misclassification, biasing effect estimates toward the null. This limitation is particularly relevant for the null findings observed for TNBC, where insufficient exposure characterization may have attenuated true subtype-specific associations.

The molecular and translational evidence base remains relatively sparse and heterogeneous. The four studies included in this review differed in design, platforms, analytic pipelines, and definitions of smoking categories, and sample sizes were often modest [15–17, 26]. As a result, their findings, although broadly convergent, require replication in larger and more uniformly characterized cohorts. It also remains unclear to what extent the observed molecular differences reflect direct effects of tobacco carcinogens on breast tissue vs. correlated exposures or selection effects related to who continues to smoke. Finally, as with most systematic reviews, we cannot exclude publication bias, particularly for molecular and prognostic studies where null or inconclusive findings may be less likely to be published. Formal assessment of small-study effects was not feasible because no meta-analysis included ten or more studies. In addition, variability in subtype classification across studies—particularly differences in Ki-67 cut-offs, definitions of luminal A-like and luminal B-like tumors, and grouping of HER2-positive disease—may have contributed to heterogeneity in the observed associations. Immunohistochemistry-based surrogate classifications are not fully equivalent to gene-expression-based subtyping and may result in misclassification of biologically distinct entities. Such inconsistencies could attenuate true subtype-specific associations or lead to differential misclassification, particularly in analyses distinguishing luminal subtypes or HER2-positive disease

Although no language restrictions were applied during the database searches, only English-language articles were included in the final analysis, which may have introduced language bias and limited the inclusion of relevant studies from non-English-speaking regions. In addition, minor discrepancies in database-specific record counts may reflect differences in indexing practices and overlap between databases, which could have influenced the initial identification of studies.

### Implications for future research

Future research should prioritize large, well-designed prospective cohorts and case-control studies with detailed, time-updated smoking histories, consistent subtype classification using contemporary molecular assays, and rigorous adjustment for confounders. Integrating germline susceptibility, polygenic risk scores, and Mendelian randomization approaches could help clarify the causal nature and subtype specificity of smoking-breast cancer associations. On the translational side, multi-omics profiling of breast tumors from smokers and non-smokers, ideally with matched normal tissue and longitudinal sampling, is needed to delineate which smoking-related molecular alterations are true driver events and how they influence treatment response and long-term outcomes.

### Conclusions

This systematic review suggests that active cigarette smoking may be associated with a possible modest increase in the risk of hormone receptor-positive and luminal breast cancer; however, this association did not reach statistical significance and should be interpreted with caution. At the same time, emerging molecular evidence indicates that smoking may shape breast cancer biology through epigenetic remodeling, increased mutational burden, receptor instability, and shifts toward more aggressive phenotypes, providing a mechanistic framework for the generally poorer outcomes observed in smokers. Although prognostic effects vary by study population, intrinsic subtype, and treatment context, the overall pattern supports smoking as an adverse modifier of tumor behaviour and survival in several clinically relevant subgroups. These findings underscore the importance of systematic smoking assessment and cessation support in both prevention and survivorship care, and highlight the need for large, well-designed studies integrating detailed exposure data with contemporary molecular profiling to refine subtype-specific risk estimates and elucidate the biological pathways linking tobacco exposure with breast carcinogenesis and progression.

## Abbreviations

ASCO: American Society of Clinical Oncology
CIs: confidence intervals
ER: estrogen receptor
ESMO: European Society for Medical Oncology
HER2: human epidermal growth factor receptor 2
NOS: Newcastle-Ottawa Scale
PR: progesterone receptor
PRISMA: Preferred Reporting Items for Systematic Reviews and Meta-Analyses
RRs: relative risks
SABCS: San Antonio Breast Cancer Symposium
TCGA: The Cancer Genome Atlas
TNBC: triple-negative breast cancer

## Supplementary materials

The supplementary materials for this article are available at: https://www.explorationpub.com/uploads/Article/file/1002376_sup_1.pdf.

## Declarations

### Availability of data and materials

The datasets supporting the findings of this study are available from the corresponding author upon reasonable request.

### Copyright

© The Author(s) 2026.
