# Supplementary material for: Impact of smoking on subtypes and molecular profile of breast cancer: a systematic review
Source: Explor Target Antitumor Ther. 2026 Jun 16;7:1002376. doi: 10.37349/etat.2026.1002376 (PMC13341265; doi:10.37349/etat.2026.1002376)
Supplement: Supplementary file 1 [file 1002376_sup_1.pdf]

Part 1

1. PubMed (MEDLINE via PubMed)

("Breast Neoplasms"[Mesh] OR "breast cancer"[tiab] OR "mammary carcinoma"[tiab] OR "breast tumour"[tiab] OR "breast tumor"[tiab]) AND ("Smoking"[Mesh] OR smoking[tiab] OR smoker*[tiab] OR "cigarette*"[tiab] OR "tobacco"[tiab] OR "tobacco smoke"[tiab] OR "tobacco use"[tiab]) AND ("Receptors, Estrogen"[Mesh] OR "Receptors, Progesterone"[Mesh] OR "Receptor, erbB-2"[Mesh] OR estrogen receptor*[tiab] OR progesterone receptor*[tiab] OR "HER2"[tiab] OR "HER-2"[tiab] OR "triple negative"[tiab] OR "triple-negative"[tiab] OR "basal-like"[tiab] OR luminal[tiab] OR "molecular subtype*"[tiab] OR "gene expression"[tiab] OR "DNA methylation"[tiab] OR epigenetic*[tiab] OR transcriptomic*[tiab] OR "multi-omics"[tiab])

2. Embase (via Elsevier)

('breast cancer'/exp OR 'breast tumor':ti,ab OR 'breast tumour':ti,ab OR 'mammary carcinoma':ti,ab OR 'breast cancer':ti,ab)

AND

('smoking'/exp OR smoking:ti,ab OR smoker*:ti,ab OR 'cigarette'/exp OR cigarette*:ti,ab OR tobacco:ti,ab OR 'tobacco smoke':ti,ab OR 'tobacco use':ti,ab)

AND

('estrogen receptor'/exp OR 'progesterone receptor'/exp OR 'her2 receptor'/exp OR 'human epidermal growth factor receptor 2':ti,ab OR HER2:ti,ab OR 'HER-2':ti,ab OR 'triple negative':ti,ab OR 'triple-negative':ti,ab OR 'basal-like':ti,ab OR luminal:ti,ab OR 'molecular subtype*':ti,ab OR 'gene expression'/exp OR 'dna methylation'/exp OR epigenetic*:ti,ab OR transcriptomic*:ti,ab OR 'multi omics':ti,ab)

3. Scopus

(TITLE-ABS-KEY("breast cancer" OR "breast tumour" OR "breast tumor" OR "mammary carcinoma"))

AND

(TITLE-ABS-KEY(smoking OR smoker* OR cigarette* OR tobacco OR "tobacco smoke" OR "tobacco use"))

AND

(TITLE-ABS-KEY("estrogen receptor" OR "progesterone receptor" OR HER2 OR "HER-2" OR "triple negative" OR "basal-like" OR luminal OR "molecular subtype*" OR "gene expression" OR "DNA methylation" OR epigenetic* OR transcriptomic* OR "multi-omics"))

4. Web of Science Core Collection

TS=("breast cancer" OR "breast tumour" OR "breast tumor" OR "mammary carcinoma")

AND

TS=(smoking OR smoker* OR cigarette* OR tobacco OR "tobacco smoke" OR "tobacco use")

AND
TS=("estrogen receptor" OR "progesterone receptor" OR HER2 OR "HER-2" OR "triple negative" OR "basal-like" OR luminal OR "molecular subtype*" OR "gene expression" OR "DNA methylation" OR epigenetic* OR transcriptomic* OR "multi-omics")
Add one final line in the supplementary:
No date or language restrictions were applied. Searches were conducted up to November 2025.

Part 2

Table S1. NOS Assessment Table (19 Studies).

#	Study	Selection (0–4★)	Comparability (0–2★)	Outcome/Exposure (0–3★)	Total (0–9★)	Overall RoB
1	Kabat et al., 2011	4★	2★	3★	9★	Low
2	Kawai et al., 2014	4★	2★	2★	8★	Low
3	Butler et al., 2016	4★	2★	2★	8★	Low
4	Park et al., 2016	4★	2★	2★	8★	Low
5	Ellingjord-Dale et al., 2017	4★	2★	2★	8★	Low
6	Jerônimo & Weller, 2017	4★	1★	3★	8★	Low
7	Gomes et al., 2022	4★	2★	3★	9★	Low
8	Ihenacho et al., 2022	4★	2★	2★	8★	Low
9	Peñalver-Argüeso et al., 2023	4★	2★	2★	8★	Low
10	Callahan et al., 2019	2★	1★	2★	5★	Moderate
11	Takada et al., 2020	4★	0★	2★	6★	Moderate
12	Wang et al., 2021	3★	2★	2★	7★	Low
13	Ferreira et al., 2024	4★	0★	3★	7★	Low

14	Andres et al., 2015	4★	2★	3★	9★	Low
15	Goldvaser et al., 2017	4★	2★	3★	9★	Low
16	Loroña et al., 2024	4★	2★	2★	8★	Low
17	Persson et al., 2016	4★	2★	3★	9★	Low
18	Schmidt et al., 2019	4★	0★	3★	7★	Low
19	Seibold et al., 2014	4★	2★	3★	9★	Low

The Newcastle–Ottawa Scale (NOS) was used to assess the risk of bias of included observational studies. The NOS evaluates three domains: (i) selection of study groups (maximum 4 stars), (ii) comparability of groups (maximum 2 stars), and (iii) ascertainment of exposure or outcome (maximum 3 stars), yielding a total score ranging from 0 to 9 stars. Higher scores indicate lower risk of bias.

In this review, studies with 7–9 stars were considered at low risk of bias, while those with 5–6 stars were classified as moderate risk of bias; no studies were categorized as high risk (≤4 stars). The star system reflects the methodological quality within each domain, with more stars indicating better design and lower susceptibility to bias.

Study quality was considered qualitatively in the interpretation of findings, particularly when assessing the consistency of results across studies; however, no studies were excluded or weighted in the meta-analysis based on NOS score.
